# A Genome-Wide Prediction and Identification of Intergenic Small RNAs by Comparative Analysis in *Mesorhizobium huakuii* 7653R

**DOI:** 10.3389/fmicb.2017.01730

**Published:** 2017-09-08

**Authors:** Xie Fuli, Zhao Wenlong, Wang Xiao, Zhang Jing, Hao Baohai, Zou Zhengzheng, Ma Bin-Guang, Li Youguo

**Affiliations:** State Key Laboratory of Agricultural Microbiology, Huazhong Agricultural University Wuhan, China

**Keywords:** small RNAs, comparative analysis, RNA-seq, Northern blotting, *Mesorhizobium huakuii*

## Abstract

In bacteria, small non-coding RNAs (sRNAs) are critical regulators of cellular adaptation to changes in metabolism, physiology, or the external environment. In the last decade, more than 2000 of sRNA families have been reported in the Rfam database and have been shown to exert various regulatory functions in bacterial transcription and translation. However, little is known about sRNAs and their functions in *Mesorhizobium*. Here, we predicted putative sRNAs in the intergenic regions (IGRs) of *M. huakuii* 7653R by genome-wide comparisons with four related Mesorhizobial strains. The expression and transcribed regions of candidate sRNAs were analyzed using a set of high-throughput RNA deep sequencing data. In all, 39 candidate sRNAs were found, with 5 located in the symbiotic megaplasmids and 34 in the chromosome of *M. huakuii* 7653R. Of these, 24 were annotated as functional sRNAs in the Rfam database and 15 were recognized as putative novel sRNAs. The expression of nine selected sRNAs was confirmed by Northern blotting, and most of the nine selected sRNAs were highly expressed in 28 dpi nodules and under symbiosis-mimicking conditions. For those putative novel sRNAs, functional categorizations of their target genes were performed by analyzing the enriched GO terms. In addition, MH_s15 was shown to be an abundant and conserved sRNA.

## Introduction

Small non-coding RNAs (sRNAs) in bacteria are usually 50–500 nucleotides (nts) in length and fulfill their riboregulations together with Hfq (an RNA chaperone) and ribonucleases (RNases) or RNase-containing complexes to mediate the expression and stability of target mRNAs (Repoila and Darfeuille, 2009; Richards and Vanderpool, 2011; Hoe et al., 2013; Morita et al., 2015). With advances in bio-computational and experimental methods, increasing numbers of sRNAs have been found and identified in bacteria (Sridhar and Gunasekaran, 2013; Becker et al., 2014; Stubben et al., 2014; Tsai et al., 2015).

Most sRNAs studies have been carried out in Gram-negative bacteria and have mainly focused on the survey and characterization of trans-encoded RNAs (del Val et al., 2012; Robledo et al., 2015b). sRNAs contain at least two conserved stem-loop structures (Man et al., 2011; Richards and Vanderpool, 2011; Storz et al., 2011; Gottesman and Storz, 2015). Depending on their specific stem-loop structures, sRNAs can set up partial or complete base-pairing with target mRNAs to modulate mRNA posttranscriptionally or can act by sequestering protein (Backofen and Hess, 2010; Bobrovskyy et al., 2015; Schu et al., 2015; Wagner and Romby, 2015; Wang et al., 2015). Alternative stem-loop structures allow sRNAs to combine with different effect factors, such as coenzymes, purines, amino acids and other compounds, and which then cause responses to changes in cell's physiology or environment (Wilderman et al., 2004; Guillier and Gottesman, 2008; Beisel and Storz, 2010; Storz et al., 2011). Moreover, in most trans-encoded sRNAs, the basically consensus Hfq-binding signatures and anti-Shine-Dalgarno sequences were inferred from covariance models in each sRNA family (Monteiro et al., 2012; Sobrero and Valverde, 2012; Wilms et al., 2012a). sRNAs regulate diverse processes, including carbon metabolism, quorum sensing, cell division, virulence, iron uptake, oxidative stress, heat shock and antibiotic resistance so on (Valverde et al., 2008; Berghoff et al., 2009; Repoila and Darfeuille, 2009; Stubben et al., 2014; Wagner and Romby, 2015; Baumgardt et al., 2016). Recently, A nodule formation efficiency sRNA NfeR1 from *S. meliloti* was reported to effect on osmoadaptation and symbiotic efficiency of rhizobia (Robledo et al., 2017). Moreover, a trans-sRNA EcpR1 was revealed to be broadly conserved in Rhizobiales and to contribute to the modulation of cell cycle regulation under detrimental conditions (Robledo et al., 2015a). MmgR is another trans-encoded small RNA, and highly conserved among the a-proteobacteria. MmgR regulates the cellular Polyhydroxybutyrate accumulation and is controlled by the cellular nitrogen status in *S. meliloti* (Ceizel Borella et al., 2016; Lagares et al., 2016, 2017). In cyanobacteria, a nitrogen stress-induced RNA (NsiR4) was shown to be involved in the regulation of glutamine synthetase, a key enzyme required for biological nitrogen assimilation (Klahn et al., 2015).

In recent decades, many bio-computational methods have been developed to predict bacterial sRNAs. The RNA sequence homology, the thermodynamically favorable secondary structure, and the conserved and consensus secondary structures are the initial parameters that are commonly adopted to predict bacterial sRNAs in intergenic genomic regions (IGRs), which can be scanned by using software such as QRNA, RNALfold and RNAz (del Val et al., 2007; Livny, 2007; Livny and Waldor, 2007). Subsequently, more complex combined approaches have been used to predict sRNAs by comparative genomics. More recently, transcriptional signs-based methods that include the prediction of transcription factor binding sites, promoters and terminator signals have changed the focus toward the predicting bacterial genomic transcription units (Chang et al., 2010; Pellin et al., 2012; Sridhar and Gunasekaran, 2013; Su et al., 2014; Tsai et al., 2015).

In rhizobia, *Sinorhizobium meliloti* is the first strain in which sRNAs in IGRs were screened by comparative genomic sequences from eight related alpha-Proteobacteria using the programs eQRNA and RNAz programs as predictive tools. Eight of the original 32 candidates were confirmed to express small transcripts by using Northern blotting experiments (del Val et al., 2007). Subsequent comprehensive genome-wide screening and identification of sRNAs were carried out in a few species of Rhizobials, including *Sinorhizobium meliloti, Rhizobium etli* and *Bradyrhizobium japonicum* (Ulve et al., 2007; Valverde et al., 2008; Voss et al., 2009; Schluter et al., 2010; Vercruysse et al., 2010; Madhugiri et al., 2012; Becker et al., 2014; Lopez-Leal et al., 2014; Hahn et al., 2016). *Mesorhizobium huakuii* is a Gram-negative bacterium that belongs to the Rhizobials of alpha-Proteobacteria. *M. huakuii* interacts with its specific host plant, *Astragalus sinicus* L., and performs nitrogen-fixation by forming indeterminate-type nodules. Recently, the sequencing of the entire genome of *M. huakuii* 7653R was completed. Its genome was found to be composed of a chromosome (6,364,365 bp), and two megaplasmids, pMhu7653Ra (193,835 bp) and pMhu7653Rb (323,475 bp), with 7,205 protein-coding genes (Wang et al., 2014). Subsequently, the transcriptomes of *M. huakuii* 7653R in bacteroids and free-living cells were analyzed and compared using RNA-seq and microarrays. However, due to the low sequencing depth, the detected genes were limited to mRNAs (≥200 bp)(Peng et al., 2014). In this study, we predicted the existence of novel sRNA genes in the IGRs of *M. huakuii* 7653R via genome-wide comparisons with four related mesorhizobial strains, including *M. huakuii* bv. *loti* MAFF303099, *M. ciceri* bv. *biserrulae* WSM1271, *M. australicum* WSM2073 and *M. opportunistum* WSM2075. The transcription units of the predicted sRNAs were further analyzed basing on the high-throughput deep sequencing data of the *Mesorhizobium huakuii* 7653R global transcriptome, and the expression profiles of the nine selected sRNAs under diverse stress conditions were revealed using Northern blotting.

## Results

### Prediction of potential sRNAs in the IGRs of *M. huakuii* 7653R

The IGRs of *M. huakuii* 7653R with a length ≥50 nt are 5125, and these were compared with four other *Mesorhizobial* strains by WU-BLASTN to identify conserved candidate sequences in the IGRs. The resulting homologies of IGRs revealed by these genome-wide comparisons were further analyzed. Raw data from ~1,500 sequences were detected after WU-BLAST analysis. Each of the conserved intergenic regions in these candidate sequences was scanned individually using eQRNA and RNAz, and overlapping sequences were determined. Meanwhile, the promoters and Rho-independent terminators were predicted using Promoter 2.0, RNAMotif and Erpin, and the resulting information was used to further assess the sequences mentioned above (Tables S1, S2). Finally, 40 conserved sequences were identified as potential sRNAs (Table 1).

**Table 1:** The candidate sRNAs in the IGRs of *M. huakuii* 7653R and the annotated functional sRNAs deduced by comparing the predicted sRNAs of 7653R with annotated sRNAs in the Rfam database.

**Candidate**	**sRNA strand**	**Predicted start**	**Predicted end**	**Predicted length**	**IGR_id**	**Flanking genes**	**Sequence start**	**Sequence end**	**Sequence length**	**Gene**
MH_s1	–	2600	2657	58	IGR_Pa-3	*MCHK_RS30880*(+)/ *MCHK_RS30885*(+)	2599 (2601/+)^**^	2660 (2727/+)	62 (127)	RF262852|AP003017, ctRNA
MH_s2	+	4846	4927	82	IGR_Pa-5	*MCHK_RS30885*(+)/ *MCHK_RS30890*(−)	4833	4906	74	RF401853|AE008135, AE009169; suhB
MH_s3^*^	−	61072	61423	352	IGR_Pa-50	*MCHK_RS31140*(−)/ *MCHK_RS31145*(−)	61074	61262	189	un
MH_s4	−	2432	2496	65	IGR_Pb-1	*MCHK_RS31790*(+)/ *MCHK_RS31795*(+)	2418 (2344/+)^**^	2515 (2474/+)	98 (131)	RF262852|AP003017, ctRNA
MH_s5	+	27793	28144	352	IGR_Pb-19	*MCHK_RS31915*(+)/ *MCHK_RS31920*(+)	27704	28144	441	RF01793, ffh
MH_s6	−	58029	58284	256	IGR_G-46	*MCHK_RS00260*(−)/ *MCHK_RS00265*(+)	no	no	no	un
MH_s7^*^	−	72421	72732	312	IGR_G-58	*MCHK_RS00320*(−)/ *MCHK_RS00325*(+)	72492	72661	170	un
MH_s8	−	866178	866302	125	IGR_G-605	*MCHK_RS03825*(−)/ *MCHK_RS03830*(−)	866178	866302	125	RF00050; FMN
MH_s9	−	1019673	1019854	182	IGR_G-732	*MCHK_RS04550*(+)/ *MCHK_RS04555*(+)	no	no	no	RF00174, Cobalamin, riboswitch
MH_s10^*^	+	1235498	1235640	143	IGR_G-893	*MCHK_RS05520*(−)/ *MCHK_RS05525*(+)	1235474	1235614	141	un
MH_s11^*^	+	1431996	1432205	210	IGR_G-1069	*MCHK_RS06525*(+)/ *MCHK_RS06530*(−)	1432008	1432172	165	un
MH_s12	+	1559635	1559846	212	IGR_G-1203	*MCHK_RS07310*(+)/ *MCHK_RS07315*(+)	1559651	1559735	85	un
MH_s13	+	2052016	2052131	116	IGR_G-1629	*MCHK_RS09840*(−)/ *MCHK_RS09845*(+)	2051956 (2052083/+)^**^	2052321 (2052213/+)	366 (131)	un
MH_s14	+	2507050	2507158	109	IGR_G-2003	*MCHK_RS12070*(−)/ *MCHK_RS12075*(+)	2506941	2507200	260	RF00504, glycine riboswitch
MH_s15^*^	−	2726638	2726908	271	IGR_G-2184	*MCHK_RS13135*(−)/ *MCHK_RS13140*(−)	2726658	2726861	204	un
MH_s16	−	2796896	2797053	158	IGR_G-2240	*MCHK_RS13460*(+)/ *MCHK_RS13465* (−)	2796947	2797069	123	un
MH_s17	−	2884712	2884910	199	IGR_G-2307	*MCHK_RS13870*(−)/ *MCHK_RS13875*(+)	no	no	no	RF00174, Cobalamin, riboswitch
MH_s18	+	2932002	2932224	223	IGR_G-2341	*MCHK_RS14095*(+)/ *MCHK_RS14100*(+)	2931996	2932217	222	Cobalamin(maybe)
MH_s19	−	3053514	3053641	128	IGR_G-2430	*MCHK_RS14720*(−)/ *MCHK_RS14725*(+)	3053513	3053602	90	RF01849, alpha_tmRNA
MH_s20	−	3136500	3136900	401	IGR_G-2487	*MCHK_RS15095*(−)/ *MCHK_RS15100*(−)	3136500	3136902	403	RF00010, RNaseP
MH_s21	−	3299281	3299382	102	IGR_G-2605	*MCHK_RS15825*(−)/ *MCHK_RS15830*(+)	no	no	no	RF0059, TPP riboswitch
MH_s22^*^	+	3491673	3491835	163	IGR_G-2770	*MCHK_RS16785*(+)/ *MCHK_RS16795*(−)	3491690	3491875	186	un
MH_s23	−	4347901	4348213	313	IGR_G-3495	*MCHK_RS21045*(−)/ *MCHK_RS21050*(+)	4347914	4348238	325	RF00518, speF
MH_s24	+	4403151	4403231	81	IGR_G-3535	*MCHK_RS21275*(−)/ *MCHK_RS21280*(−)	4403128	4403198	71	RF00519, suhB
MH_s25^*^	−	4532142	4532343	202	IGR_G-3642	*MCHK_RS21925*(+)/ *MCHK_RS21930*(+)	4532144 (4531972/−)^**^	4532330 (4532239/−)	187 (268)	un
MH_s26	−	4579379	4579594	216	IGR_G-3682	*MCHK_RS22185*(−)/ *MCHK_RS22190*(+)	4579346	4579554	209	un
MH_s27	+	4677352	4677482	131	IGR_G-3753	*MCHK_RS22640*(−)/ *MCHK_RS22645*(−)	4677314	4677396	83	RF01118, snmRNA
MH_s28	−	4681680	4681810	131	IGR_G-3754	*MCHK_RS22655*(−)/ *MCHK_RS22660*(−)	4681648	4681722	75	n3555, AJ544053, snmRNA
MH_s29	+	4780322	4780399	78	IGR_G-3840	*MCHK_RS23145*(−)/ *MCHK_RS23150*(+)	4780323	4780503	181	RF00521, SAM_alpha
MH_s30	−	5045563	5045615	53	IGR_G-4067	*MCHK_RS24485*(−)/ *MCHK_RS24490*(−)	5045561	5045692	132	RF00517, SerC
MH_s31	−	5068059	5068203	145	IGR_G-4090	*MCHK_RS24605*(−)/ *MCHK_RS24610*(−)	5068057	5068160	104	un
MH_s32	−	5092499	5092668	170	IGR_G-4120	*MCHK_RS24760*(−)/ *MCHK_RS24765*(+)	5092499	5092638	140	RF00013, 6S RNA
MH_s33	−	5178956	5179352	397	IGR_G-4190	*MCHK_RS25165*(−)/ *MCHK_RS25170*(−)	5178945	5179159	215	RF01867, CC2171
MH_s34	−	5355054	5355121	68	IGR_G-4348	*MCHK_RS26065*(−)/ *MCHK_RS26070*(−)	5355042	5355141	100	RF00520, ybhL
MH_s35	+	5723839	5723889	51	IGR_G-4638	*MCHK_RS27895*(−)/ *MCHK_RS27900*(+)	5723811	5723946	135	RF01068, mini-ykkC
MH_s36^*^	−	5779014	5779103	90	IGR_G-4677	*MCHK_RS28170*(−)/ *MCHK_RS28175*(−)	5779015	5779087	73	un
MH_s37	−	5845302	5845459	158	IGR_G-4735	*MCHK_RS28500*(−)/ *MCHK_RS28505*(−)	5845359	5845423	65	un
MH_s38	+	5936318	5936708	391	IGR_G-4809	*MCHK_RS28940*(−)/ *MCHK_RS28945*(+)	no	no	no	RF00174, Cobalamin_riboswitch
MH_s39^*^	+	6026827	6027187	361	IGR_G-4878	*MCHK_RS29340*(−)/ *MCHK_RS29345*(−)	6026852	6027101	250	un
MH_s40	+	6301829	6302173	345	IGR_G-5080	*MCHK_RS30580*(−)/ *MCHK_RS30585*(+)	6301885	6302173	289	RF00169, Bacteria small SRP, 4.5S

* *sRNAs were selected for Northern blotting*.

** *transcription was detected in the opposite strand or additional transcription was found in the region of the predicted sRNA*.

“*no” indicates that the predicted sRNA was not detected in sequencing products*.

“*un” indicates that the candidate sRNA was not annotated in the Rfam database and its function is unknown*.

To find the annotated known sRNAs in 7653R, we performed Rfam searches using the above potential sRNAs. Of the 40 predicted sRNAs, 24 were annotated in the Rfam database, thus, the other 16 sRNAs are regarded as putative novel sRNAs (Table 1). Several known sRNAs, which are highly conserved in bacteria, were successfully predicted, including 6S RNA (MH_32), 4.5S (MH_s40), RNase P class A (MH_s20), and tmRNA (MH_s19), suggesting that the methods used in this study worked well. A few glycine, cobalamin and TPP riboswitches were also detected. Notably, most of the sRNAs (35 of 40) were located on the chromosome, with only a few (three in pMhu7653Ra and two in pMhu7653Rb) located on megaplasmids, indicating possible differential conservation of the IGR sequences between the chromosome and megaplasmids among the tested bacteria. Predicting the sRNAs that locate on the symbiotic megaplasmids seems to be difficult.

### Predicted sRNA transcription units

As noted above, the genomic comparison identified 40 potential sRNAs. Further analyses were conducted to describe their transcription units and transcriptional start regions using Illumina high-throughput sequencing data. These analyses were performed on RNA isolated from 28- and 50-dpi (days post-infection) symbiotic nodules and free-living cells of 7653R (see Materials and Methods) that were treated with TAP. The depths of RNA-seq were shown in Tables S3, S4.

A comparison of the analysis between the predicted sRNAs and RNA-seq products showed that, apart from 5 riboswitches, 19 functional annotated sRNAs and 15 of 16 putative novel sRNAs were detected in the sequencing dataset, and only one was not expressed in plant nodules or in free-living cultured cells (Table S5). Of the riboswitches (cis-regulators of mRNA that control the translation of mRNA), MH_s14, was observed to be highly expressed in all sequenced samples. In total, other than the 5 riboswitches, 34 of the 35 predicted sRNAs had corresponding RNA-seq sequencing products, and only MH_s6 was absent in the sequenced samples. These results suggest that MH_s6 expression might be induced by different stress conditions rather than the logarithmic growth state or symbiosis or that the MH_s6 transcript levels were below the threshold of detection using RNA-seq. Notably, except for a few sRNAs (MH_s1, MH_8, MH_s10, and MH_s20), the lengths of the sequenced sRNAs in the RNA-seq data were slightly longer (10 of 34 sRNAs) or shorter (20 of 34 sRNAs) than the predicted lengths. The predicted transcriptional start and stop regions of the sRNAs were also compared with the RNA-seq data, and the results showed that 12 and 5 predicted sRNAs had approximate matches to their sequenced start and stop positions, respectively. Thus, more than 50% of the predicted sRNAs showed transcript length discrepancies with their RNA-seq products. This result has also been observed by others (Gonzalez et al., 2008; Valverde et al., 2008; Otto et al., 2012; Mentz et al., 2013), and the main reason may be ascribed to RNA processing or degradation in the cell (Madhugiri et al., 2012; Saramago et al., 2014).

### Annotation and distribution of known conserved sRNAs

**Figure 1:**
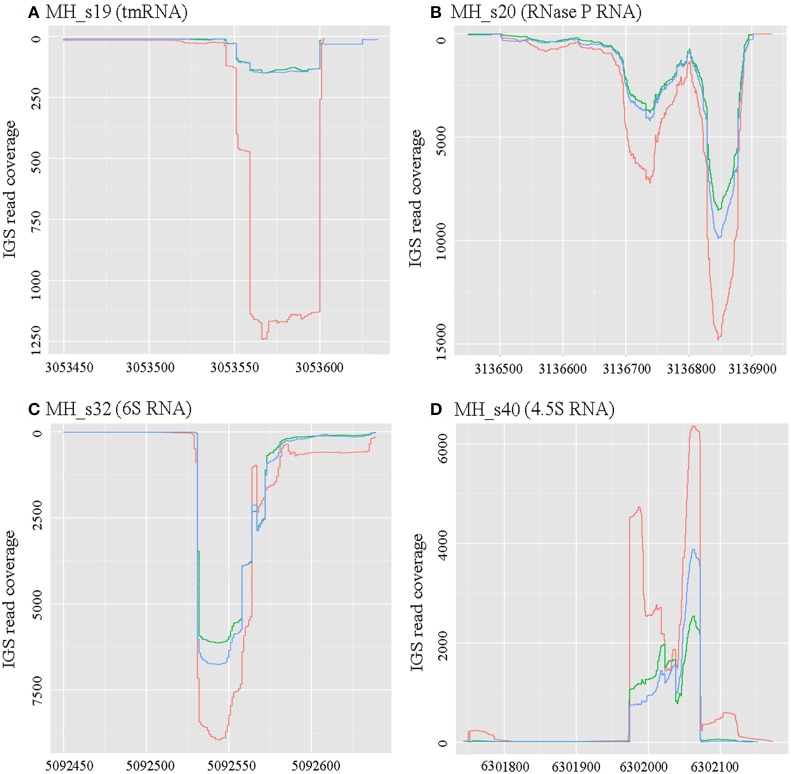
Expression profiles of four known conserved small RNAs. RNAseq raw read coverage traces of each sRNA are shown and were derived from the RNAseq libraries of free-living cells (red), 28-dpi (green) nodules, and 50-dpi nodules (blue). The coordinates for the x- and y-axes represent the genome alignment and coverage, respectively. **(A)** Expression profile of MH_s19 (tm RNA); **(B)** Expression profile of MH_s20 (RNase P RNA); **(C)** Expression profile of MH_s32 (6S RNA); **(D)** Expression profile of MH_s40 (4.5S RNA). In these four known conserved sRNAs, RNase P showed the highest expression levels under both free-living and symbiotic conditions.

There are four sRNAs, including 6S RNA, 4.5S RNA, bacterial RNase P class A, and tmRNA, that are highly conserved and regarded as housekeeping sRNAs in bacteria. In this study, these four housekeeping sRNAs were detected by both bioinformatic analysis and RNA-seq, and their expression profiles and expression levels are respectively shown in Figure 1 and Table 2. In agreement with the earlier reports, RNase P (MH_S20), an omnipresent endoribonuclease, was highly expressed under both free-living and symbiotic conditions and displayed the highest expression levels compared with other housekeeping sRNAs (Regalia et al., 2002; Vercruysse et al., 2010). The 4.5S RNA (MH_S40), which forms the signal recognition particle (SRP) with the Ffh protein, was also relatively highly expressed, especially in symbiotic bacteroids (~1.5-fold higher than in free-living cells). The 6S sRNA (MH_S32) has been reported to be more abundant during the stationary phase of cell growth. Its function is to block δ^70^-dependent transcription in alpha-Proteobacteria. In our sequencing results, 6S was strongly expressed in exponential-phase cells (OD_600_ = 0.8), and abundant transcripts were also detected in mature (28 dpi) and senescent (50 dpi) nodules. The expression of tmRNA (MH_S19), which rescues stalled ribosomes and tags incomplete polypeptides for degradation, was lower in both free-living and symbiotic conditions, and down-regulated in mature and senescent nodules compared with exponential-phase cells (>2-fold).

**Table 2:** The differentiation and fold-changes of known conserved sRNA expression levels under different growth conditions based on the *M. huakuii* 7653R RNA-seq data.

**Conserved sRNA**	**Candidate**	**Length**	**Sequence start and end (transcriptional direction)**	**FC(rpkm)**	**MN(rpkm)**	**SN(rpkm)**	**MN/FC**	**SN/FC**
ctRNA (pMhu7653Ra)	MH_s1	62	2599–2660(−)	1, 394.33	381.45	365.20	0.27	0.26
	MH_s1^*^	126	2602–2727(+)	101.16	50.98	60.11	0.50	0.59
ctRNA (pMhu7653Rb)	MH_s4	98	2418–2515(−)	911.45	302.92	314.35	0.33	0.34
	MH_s4^*^	131	2344–2474(+)	147.02	157.55	104.17	1.07	0.71
tmRNA	MH_s19	90	3053513–3053602(−)	163.27	72.76	70.51	0.45	0.43
RNase	MH_s20	403	3136500–3136902(−)	692.90	1, 039.98	1, 205.31	1.50	1.74
6S RNA	MH_s32	140	5092499–5092638(−)	1, 244.63	1, 253.26	1, 299.15	1.01	1.04
4.5S RNA	MH_s40	289	6301885–6302173(+)	523.54	883.04	859.06	1.69	1.64

* *Additional transcription was detected in the opposite strand*.

*FC represents free-living cells, MN represents mature nodules (28 dpi) and SN represents senescent (50 dpi) nodules. No means the gene expression in free-living cells is zero*.

**Figure 2:**
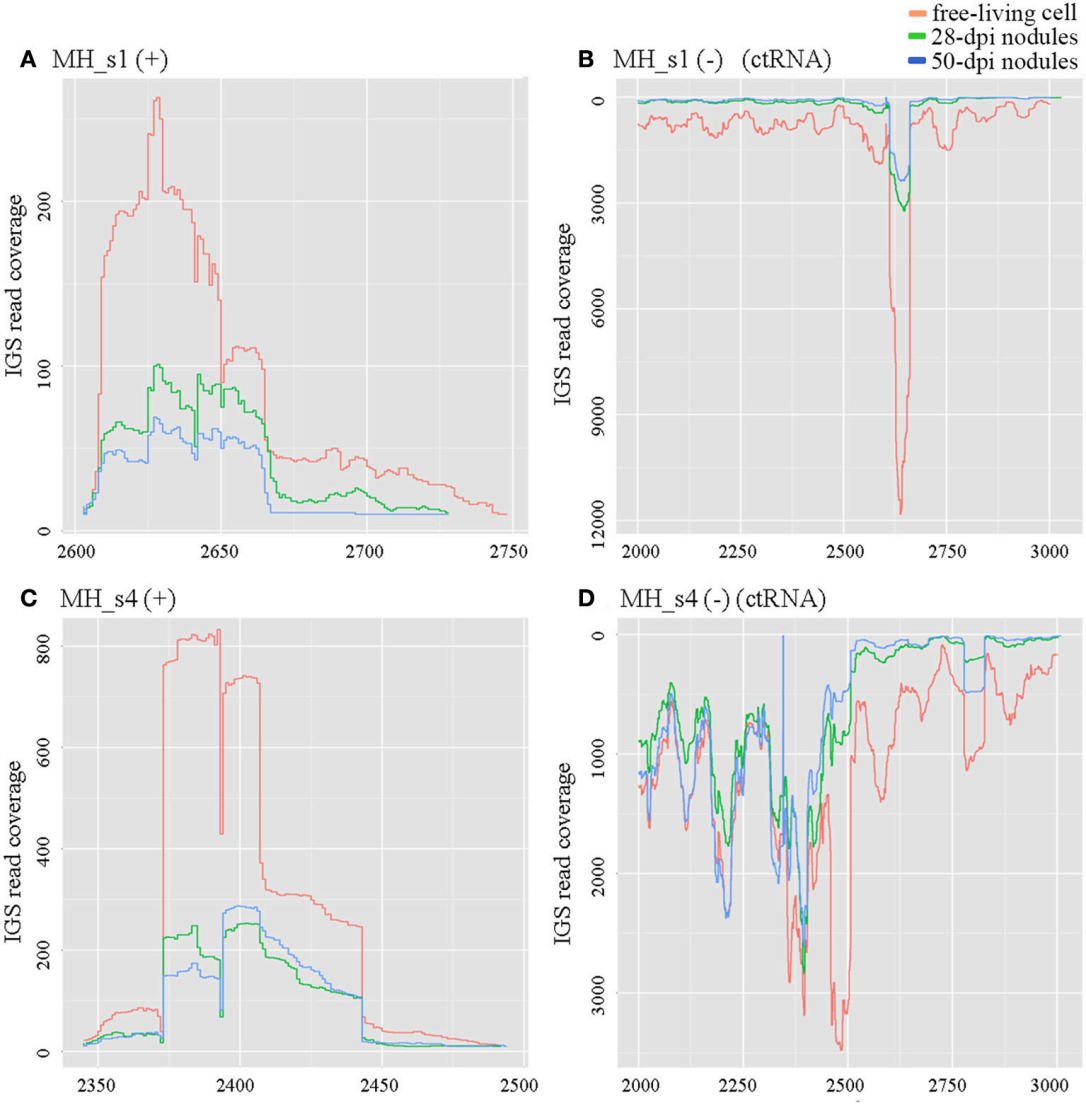
Expression profiles of ctRNA and its opposite strands in pMhu7653Ra (MH_s1) and pMhu7653Rb (MH_s4). The levels of ctRNA were very high in free-living cells in both pMhu7653Ra and pMhu7653Rb. **(A)** Expression profile of MH_s1 (+); **(B)** Expression profile of MH_s1 (−); **(C)** Expression profile of MH_s4 (+); **(D)** Expression profile of MH_s4 (−). A cis-encoded, highly-abundant sequencing product was observed in the opposite strands of ctRNA, which was located at 2,601–2,727 in pMhu7653Ra and 2,344–2,474 in pMhu7653Rb.

ctRNA (counter-transcribed RNA) is a highly conserved small RNA located in repB–repC intergenic regions. It regulates the replication and incompatibility of repABC-type plasmids (MacLellan et al., 2005; Vercruysse et al., 2010; Rivera-Urbalejo et al., 2015). *M. huakuii* 7653R possesses two symbiotic megaplasmids required for symbiosis and nitrogen fixation. In our results, the levels of ctRNA (MH_s1 in pMhu7653Ra and MH_s4 in pMhu7653Rb) were very high in free-living cells, approximately 3- to 4-fold higher in 28 dpi and 50 dpi nodules, respectively (Figure 2). Moreover, a cis-encoded highly abundant sequencing product was observed in the repB–repC intergenic regions of pMhu7653Ra (located at 2601–2727) and pMhu7653Rb (located at 2344–2474), respectively, so we speculated that this cis-encoded product could be formed by ctRNA terminating the transcription of repC. The results further showed that ctRNA is trans-transcribed and highly expressed in rhizobia (Yip et al., 2015).

### Experimental verification of nine selected sRNAs by Northern blotting

**Figure 3:**
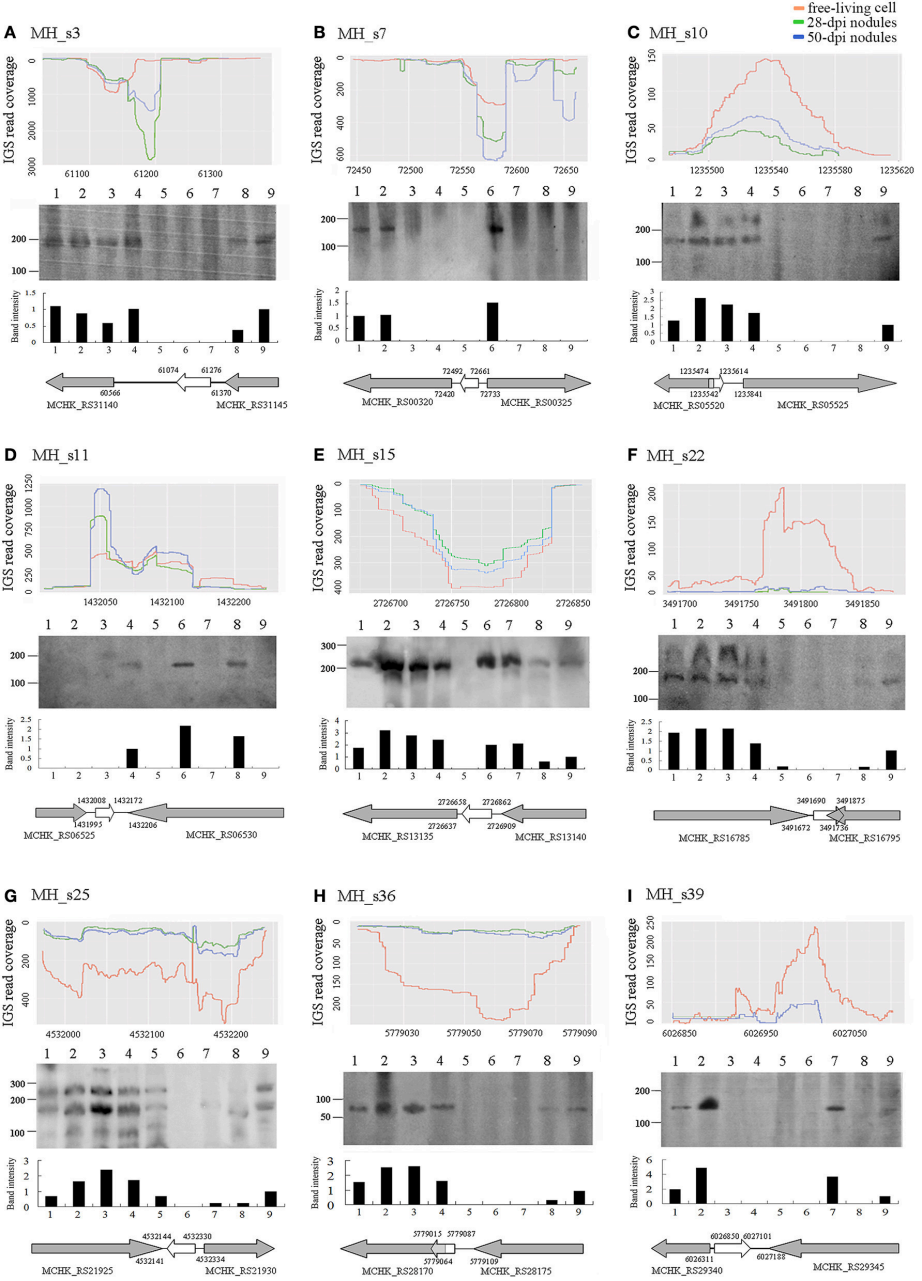
Expression profiles **(A–C)**, northern blots **(D–F)**, and transcription directions (**G–I**) of nine selected small RNAs. The expression profiles of nine selected sRNAs from the RNAseq raw reads of free-living cells, 28-dpi nodules and 50-dpi nodules are shown on the top of each column. The details are described in Figure 1. Northern blot results are shown in the middle of each column. Lanes 1–9 represent different tested conditions, which include symbiosis (lane 1, 28 dpi nodules), micro-oxygen stress (lane 2, >99% N_2_), oxidative stress (lane 3, 2 mM H_2_O_2_), salt stress (lane 4, 4 M NaCl), acid stress (lane 5, pH 5), alkali stress (lane 6, pH 9), cold (lane 7, 20°C), heat stress (lane 8, 37°C) and the unstressed condition (lane 9, log phase cells) (see Section Materials and Methods). Below the read coverage traces, small RNA gene regions and transcription directions are indicated by arrows of different lengths and colors. Gray arrows represent flanking genes and white arrows represent the sRNA genes.

To confirm the existence of the predicted novel sRNAs and to understand their basic functions, 9 of the 15 potential novel sRNAs were selected randomly and detected by conducting Northern blotting on the total RNA extracted from cultured cells grown under non-stressed and stressed conditions (Figure 3), and 5S RNA was used as a positive control (Figure S1). As shown, the sizes of the nine sRNAs that were tested roughly consistent with those of the RNA sequencing dataset. Two candidate sRNAs (MH_s10 and MH_s22) exhibited two bands, and MH_s25 showed a complex banding pattern. Six of the nine tested sRNAs (MH_3, MH_s10, MH_s15, MH_s22, MH_s25, and MH_s36) displayed strong signals in response to a variety of external stimuli. MH_s7, MH_s11 and MH_s39 were expressed under only rare growth conditions. In addition, after analyzing the sequencing products again, another highly expressed sequence (MH_s25) was detected that located between chromosomes 4531972 and 4532239. Because this transcript product lacked typical conserved structures and 4532144–4532330 is close to the predicted region (4532142–4532343), we regard MH_s25 (4532144–4532330) as a potential sRNAs. Apart from 4532144–4532330 and 4532142–4532343, the further information on other Northern blotting signals of MH_s25 could not be detected in the RNA-seq data. The main reason may be that the bio-informational analysis and compilation were performed to deal with an enormous number of non-coding RNAs in the RNA-seq data, which led to some shorter or less abundant transcripts usually be merged into the longer ones in a same transcription unit, so the sequence information for complex banding pattern appearing in Northern blots was difficult to obtain.

The expression and abundance of sRNAs were affected by various external stimuli, and these condition-specific expression patterns help to infer the functions of different sRNAs (Vercruysse et al., 2010). In our study, most of the tested sRNAs (MH_3, MH_s10, MH_s15, MH_s22, MH_s25, and MH_s36) were highly abundant in symbiotic, micro-oxygen, oxidative and salt-stress conditions, but few sRNAs accumulated under heat, cold, acid, or alkali stress conditions. MH_s15 and MH_s25 were widely expressed sRNAs and accumulated heavily under most of the tested induction conditions, suggesting that they function in multiple cellar processes. The expression patterns of MH_s7, MH_s11 and MH_s39 were unique, and these sRNAs accumulated under only a few of the tested conditions. Nearly all the detected sRNAs (8 of 9, MH_s11 being the exception) were expressed under symbiotic conditions or symbiosis-mimicking (micro-oxygen and H_2_O_2_) stimuli, indicating that a large number of sRNAs are required during rhizobial symbiotic nitrogen fixation. However, more studies are required to characterize the functions and mechanisms of action of these sRNAs.

### Conservation and structural characterization of nine identified sRNAs

**Figure 4:**
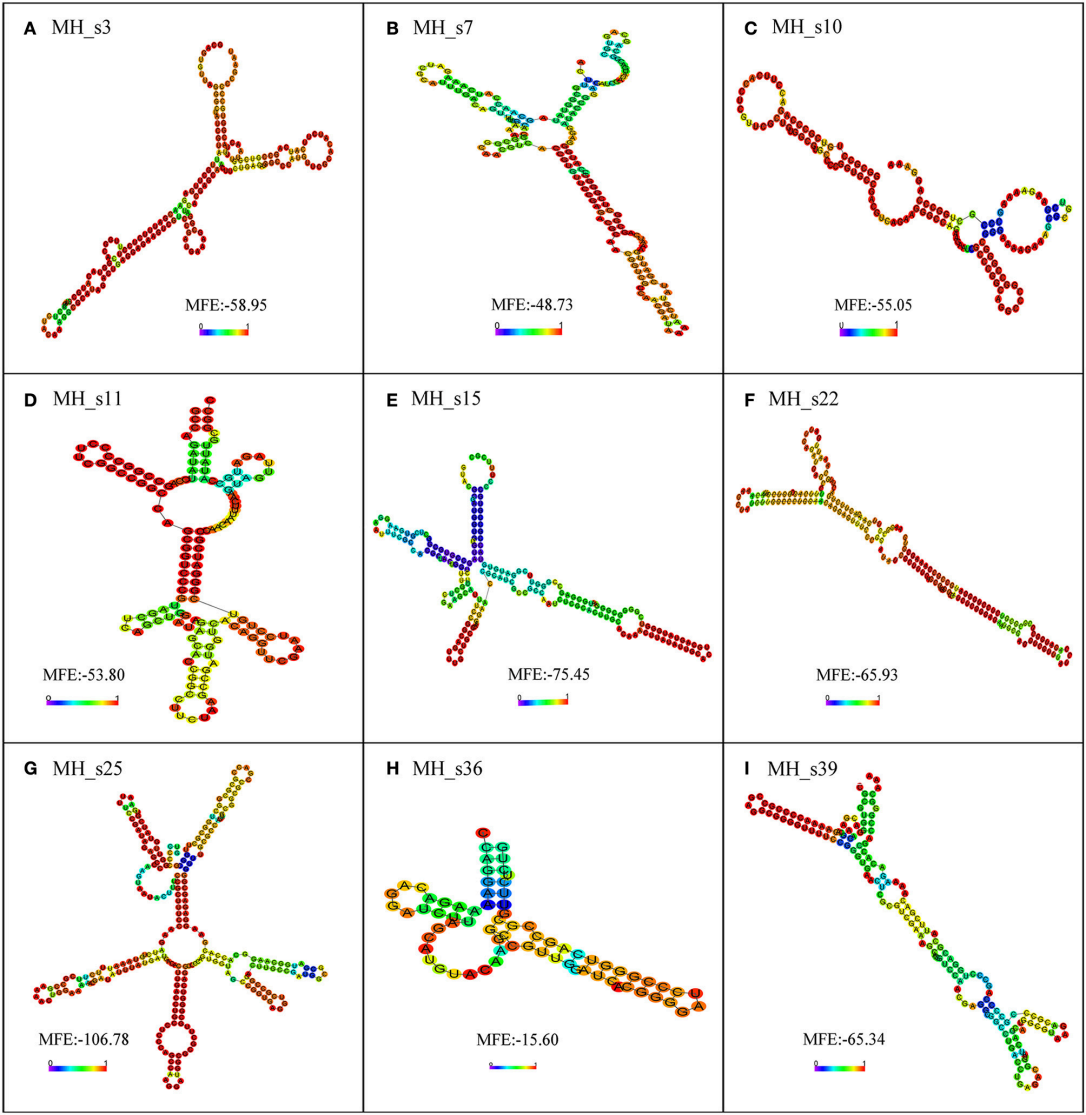
The secondary structure prediction of nine small RNAs determined by using RNA-fold. The secondary structures are colored according to the base-pairing probabilities. The unpaired regions are colored according to the probabilities of being unpaired. The lowest minimum free energy (MFE) of each sRNA, analyzed using RNA-fold, is shown in the diagram. **(A)** Secondary structure of MH_s3; **(B)** Secondary structure of MH_s7; **(C)** Secondary structure of MH_s10; **(D)** Secondary structure of MH_s11; **(E)** Secondary structure of MH_s15; **(F)** Secondary structure of MH_s22; **(G)** Secondary structure of MH_s25; **(H)** Secondary structure of MH_s36; **(I)** Secondary structure of MH_s39.

To further examine the characteristics of the detected sRNAs, a BLASTN search (NCBI) was performed to analyze the conservation of all the detected sRNAs in *M. huakuii* 7653R. The results showed that most of the detected sRNAs are highly conserved and only distributed in *Mesorhizobium* spp. and *Bradyrhizobium japonicum*, except for MH_s11, which has homologs in *Bartonella* spp. The detected sRNAs contain highly conserved secondary structures and more than two loops (Figure 4). MH_s15 has highly conserved structures, with a single, clear band shown on the Northern blots, and abundant polyA/U in the loop and tail of its secondary structure, which is the putative Hfq-binding site. In addition, the transcription levels of the genes upstream and downstream of MH_s15 were upregulated in 28 and 50 dpi nodules, respectively, compared with free-living cells (from 1.5- to 7-fold), according to the RNA-seq results. Therefore, the target genes of MH_s15 were analyzed by using the IntaRNA software and the potential conserved interactive regions were shown in Table S6, but further experiments are needed to characterize the interactions between MH_s15 and its target genes and determine the influence of MH_s15 on the symbiosis between *M. huakuii* 7653R and host.

### Target genes prediction of candidate sRNAs and functional analysis

**Figure 5:**
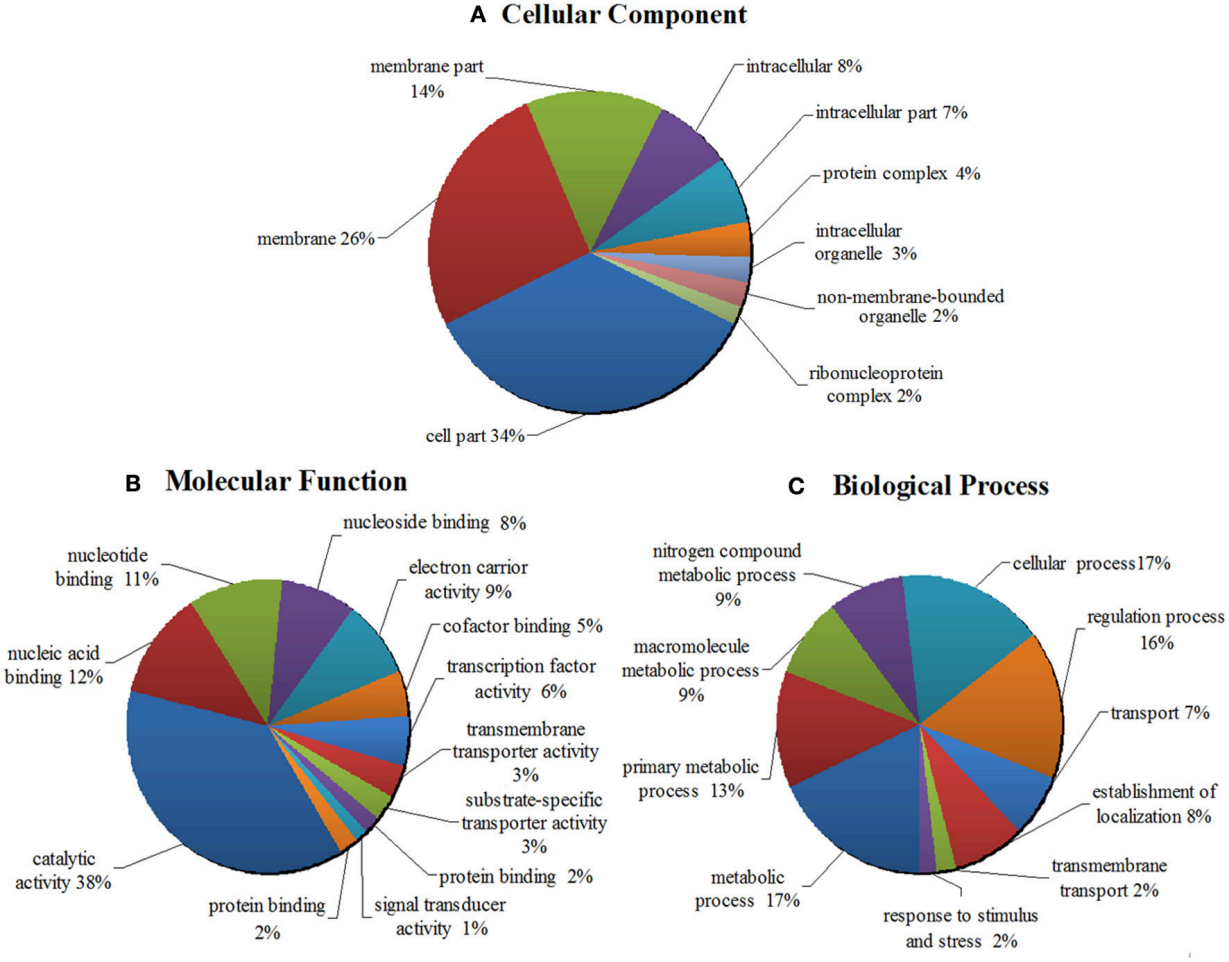
GO analyses and functional classifications of predicted target genes of 16 putative novel sRNA in *Mesorhizobium huakuii* 7653R. The target genes of sRNA candidates were predicted by using the program IntaRNA, the GO term annotations were finished by the tool of InterPro, and the program WEGO was used to classify and plot the GO annotations. The enriched GO terms and functional categorization of candidate sRNA target genes were summarized. It showed that they are involved in cellular components **(A)**, molecular functions **(B)**, and biological processes **(C)**. The target genes contained in each functional category are indicated as percentages (in brackets) of the total number of genes with GO annotations.

In order to better understand the potential function of the 16 new sRNA candidates identified in this work, the target genes of 16 putative novel sRNAs were predicted by using the webserver IntaRNA. These candidate target genes were subjective to GO annotation and enrichment analysis. The WEGO output of the target genes was shown in Figures S2, S3. The enriched GO terms and functional categorization of candidate small RNA target genes were summarized and demonstrated in a pie chart (Figure 5, Table S8). As it showed in Figure 5A, classified in terms of cellular component, a majority of the predicted target genes is enriched in the function of membrane systems (40%), cell part (34%) and intracellular part (15%). When classified as molecular functions, a total of 38% of the enriched GO terms are related to catalytic activity, and 21% of the target genes are involved in the binding of nucleic acid, nucleoside and nucleotide (Figure 5B). Regarding the enriched GO term of biological process, as shown in Figure 5C, the target genes were distributed widely in various biological functions, from cellular process, to biological regulation, to primary metabolic process, to nitrogen compound metabolic process, and to response to stimulus and stress. Importantly to point out, 48% of the target genes are enriched in all kinds of metabolic processes, such as primary metabolism, second metabolism, nitrogen compound metabolism, alcohol metabolism etc. Remarkably, 9% of the target genes are involved in the nitrogen compound metabolic process, indicating the important roles of the sRNAs playing in the nitrogen fixing and utilization. Moreover, 16 and 17% of the target genes are respectively involved in biological regulation and cellular process, and 2% of the target genes are connected with response to stimulus and stress (Figure 5C).

## Discussion

Genome-wide screens for sRNAs have mainly used bioinformatic prediction, RNA-seq, and tiling array analyses. In general, the number of non-coding transcripts is large, reaching hundreds or thousands of species in an RNA-seq or tiling array experiment (Vercruysse et al., 2010; Otto et al., 2012; Wilms et al., 2012b; Ignatov et al., 2013; Jimenez-Zurdo and Robledo, 2015). Hence, effective and accurate sRNA analysis technology is necessary to extract potential sRNA sequences from the complicated data. In this study, because most trans-encoded sRNAs are not followed by a Rho-independent terminator (Toffano-Nioche et al., 2012; Mentz et al., 2013), we focused on the conserved intergenic regions and stable secondary structures to screen for putative novel sRNAs in the IGRs of the *M. huakuii* 7653R genome. Based on the results of a comparative analysis of five related strains, we extracted 40 potential sRNAs and used RNA deep sequencing data to further identify their characteristics. Using this strategy, 24 annotated sRNAs with well-characterized functions in bacteria and 15 putative novel sRNAs were detected.

Except for five cis-regulatory riboswitches, 34 of the 35 predicted sRNAs in *M. huakuii* 7653R were consistent with the RNA-seq data. thus, the technology used for predicting sRNAs based on structural conservation is a reliable means of revealing sRNAs in bacteria. However, the method has some flaws. Apart from the generation of a small number of false positives (Sridhar and Gunasekaran, 2013), there were obvious size differences between the predicted sRNAs and the RNA-seq products which was also observed in previous articles (Tsai et al., 2015). This result may be related to the processing or degradation of sRNAs and the corresponding RNA-seq products (Arraiano et al., 2010; Vercruysse et al., 2010; Saramago et al., 2014). More importantly, the lengths of the sequenced non-coding RNAs showed some differences between free-living cells and plant nodules, where the non-coding RNAs in free-living cells were usually slightly longer than those in nodules. This result indicates that the sizes of sRNAs are influenced by the growth conditions. Moreover, the conservation of *M. huakuii* 7653R IGR sequences might result in some false judgments about sRNA transcription units. *M. huakuii* 7653R is a special case in rhizobia and forms nodules only in *A. sinicus* L. Most of the widespread sRNAs in rhizobia, e.g., Smr45C, Smr15C1/C2(AbcR1/2) and Smr7C so on, have not been discovered in 7653R. Hence, secondary structure conservation was adopted as a main predictive parameter to screen for the putative sRNAs in this study.

In our experiments, the diverse stresses were designed to stimulate the transcription of the sRNAs. Reactive oxygen (H_2_O_2_, ${\text{O}}_{2}^{-}$) is found to have a key function in a plant's defense system against pathogens. In rhizobia, H_2_O_2_ has not been detected in free-living cells or bacteroids, but H_2_O_2_ accumulation was observed in the infection stages of the symbiotic interaction and in ultrathin sections of mature 6-week-old nodules. For this reason, H_2_O_2_ played an important signaling role in early symbiotic and senescence processes, and rhizobia have an efficient antioxidant defense system to detoxify H_2_O_2_ (Herouart et al., 2002; Pauly et al., 2006; Puppo et al., 2013; Janczarek et al., 2015). During symbiotic nitrogen fixation, anoxic stress or a low oxygen concentration is required for nitrogenase activity and the expression of nitrogen-fixing genes in bacteroids. Two oxygen-responsive regulatory systems, FixLJ-FixK and RegSR-NifA, control numerous symbiotic genes in rhizobia. When the oxygen concentration was lowered from 21 to 0.5%, the number of genes controlled by the FixLJ-FixK_2_ regulatory cassette was shown to increase progressively. Moreover, NifA-dependent genes were expressed only when the oxygen concentrations was below 2% in the gas phase. Therefore, micro-oxygen and H_2_O_2_ stimuli were applied to the free-living cells to mimicked symbiotic conditions (Sciotti et al., 2003; Dixon and Kahn, 2004; Lindemann et al., 2007; Mesa et al., 2008). By Northern blotting, we further confirmed the transcription and sizes of the nine predicted sRNAs, and found that the expression of the majority of the detected sRNAs could be induced by symbiotic or symbiosis-mimicking conditions. The expression of sRNAs is usually driven by specific environmental conditions, and in turn, the function of sRNAs is relevant to the stimulus or stress (Caswell et al., 2012; Robledo et al., 2015a). Hence, the present results indicate that a large number of sRNAs are necessary to regulate the expression of genes involved in the differentiation and maturation of bacteroids during rhizobial symbiotic nitrogen fixation. Further, we predicted the target genes of 16 functional-unknown sRNAs including those 9 validated sRNAs, and sorted out the target genes through GO terms analysis by utilizing WEGO, by which a lot of useful information to figure out the potential functions of those novel sRNA candidates is provided. It was shown that a majority of the targets are involved in various metabolic or cellular biological processes, indicating that these sRNAs play fundamental roles in bacterial primary metabolism, enzyme catalytic activity, transport, replication and transcription process etc. The significant roles of the sRNAs playing in rhizobia infection, bacteroid differentiation, nitrogen metabolism and response to stresses are revealed as well in the enriched GO terms. These observations are not strange, since these biological processes actually reflect the life cycle of rhizobia in soil and host plant. For rhizobia, to carry out symbiotic nitrogen fixation in nodule, a series of signaling transduction and mutual molecular interactions between rhizobia and host plant are required. During rhizobia symbiosis and nitrogen fixation process, the size and morphology of rhizobia change significantly, which involves many cellular parts and cellular processes. Also, many transcription factors play essential roles in regulating nodule formation and rhizobia nitrogen fixation. Rhizobia have to survive within the infected host plant cells, during which it has to response to the various stress environments. Besides, symbiotic nitrogen fixation involves many transportation events regarding carbon and energy supplying and nitrogen compound assimilation.

So far, little is known about the role of sRNAs and riboregulation in the control of symbiotic plant-microbe interactions, and the only studies that have been perfo rmed have focused on *S. meliloti* (del Val et al., 2012; Becker et al., 2014; Jimenez-Zurdo and Robledo, 2015). The homologous AbcR1 and AbcR2 (also known as Sm15/16 or Sm15C1/C2) were identified as trans-acting sRNA to fine-tune nutrient uptake (Torres-Quesada et al., 2013). EcpR1 were considered to control bacterial cell cycle-related genes. The overexpression of EcpR1 leads to cell elongation, and deletion of EcpR1 reduces bacterial competitiveness, but does not influence on symbiosis in *S. meliloti* (Robledo et al., 2015a). RcsR1 regulates quorum sensing through the autoinducer synthase gene, *sinI* (Baumgardt et al., 2016), and MmgR controls the cellular Polyhydroxybutyrate (PHB) accumulation, but all these reported sRNAs are dispensable for symbiosis (Ceizel Borella et al., 2016; Lagares et al., 2016, 2017). Recently, a novel sRNA NfeR1 (nodule formation efficiency sRNA) was characterized in *S. meliloti*. NfeR1 shows high expression in salt stress and throughout the symbiotic interaction. NfeR1 is the firstly reported sRNA to contribute to infectivity, nodule formation and development, and symbiotic nitrogen-fixtion efficiency in rhizobia (Robledo et al., 2017). In this work, MH_s15 was shown to be an abundant sRNA by using Northern blot. In addition, it is worth noting that the transcription levels of the genes upstream and downstream of MH_s15 was upregulated in nodules compared with free-living cells, and an overexpression of MH_s15 resulted in a significantly defected symbiotic phenotype (data not showed). It suggested that the function of MH_s15 would be associated with symbiosis, but more experiments are needed to elucidate its function and mechanism.

In light of the strict host specificity of *M. huakuii* 7653R for *A. sinicus* L., and especially most of the sRNA identification and validation studies in endosymbiotic nitrogen- fixing bacteria have been done in species belonging to other genus but not in *Mesorhizobium*, we believe that our findings in this work provide novel insights into the sRNA regulation of symbiotic plant-microbe interactions.

## Materials and methods

### Prediction of sRNAs in the IGRs of *M. huakuii* 7653R

The comparative analysis of IGRs was carried out among *M. austrqalicum* WSM2073, *M. ciceri* WSM1271, *M. Loti* MAFF303099, *M. opportunistum* WSM2075 and *M. huakuii* 7653R. Their genomic sequences and protein annotations were downloaded from the NCBI bacterial databases (ftp://ftp.ncbi.nih.gov/genomes/Bacteria). The predictions of sRNAs were performed as previously described (del Val et al., 2007). IGRs with a length ≥50 nt were scanned and homologous regions were clustered using WU-BLASTN (http://blast.wustl.edu) in the tested genomes with *E*-values ≤ 1e^−5^. The alignments were interrogated using eQRNA version 2.0.4 (ftp://ftp.genetics.wustl.edu/pub/eddy/software/qrna.tar.Z) as described previously (del Val et al., 2007). RNAz version 0.1.1 (http://www.tbi.univie.ac.at/~wash/RNAz) was used to screen the conserved intergenic regions in these candidate genomic sequences, and overlapping sequences between eQRNA and RNAz were selected. Subsequently, pairwise alignments in both eQRNA and RNAz were calculated using RNAfold (ViennaRNA-2.1.1) to determine the stable secondary structures, with the stem-length options set to a minimum of 20 bp. The promoters and Rho-independent terminators were analyzed using Promoter 2.0, RNAMotif and Erpin to further assess the resulting sequences. Finally, the nucleotide sequences of potential sRNAs were further inspected according to the results of RNA-seq deep sequencing. Each predicted sRNA was used to query the Rfam database to capture any annotated RNA regulatory elements.

### Bacterial cultivation and plant growth conditions

*Rhizobium* strains were cultured in tryptone yeast medium (TY) or AMS medium (FAM2 minimal salts medium supplemented with 0.1% vol/vol aniline) at 28°C, and *Escherichia coli* strains were grown in Luria-Bertani (LB) at 37°C. When required, antibiotics were added at the following concentrations: ampicillin (Ap) 100 μg/ml; gentamicin (Gm) 20 μg/ml; streptomycin (Str) 20 μg/ml and kanamycin (Kan) 50 μg/ml.

In the Northern blotting analyses, free-living rhizobial cells were cultivated in TY at 28°C, and cells were harvested in the log phase (OD_600_ = 0.4–0.5). When required, the growing log cultures were subjected to the following stress conditions as described previously (Madhugiri et al., 2012; Klahn et al., 2015): oxidative stress (2 mM H_2_O_2_ for 10 min), salt stress (4 M NaCl for 30 min), acid stress (pH5 for 90 min), alkali stress (pH9 for 90 min), cold stress (20°C for 90 min) or heat stress (37°C for 90 min). For micro-oxygen stress, log phase cells growing in TY (OD_600_ = 0.4–0.5) were shifted to a low O_2_ environment by continuous sparging of the culture with purified N_2_ for 24 h (N_2_ ≥ 99%). Controls were non-stressed exponentially growing cultures. For symbiotic conditions, surface-sterilized seeds of *A. sinicus* L. cultivar XY202 were cultivated in pots filled with sterile sand and irrigated with nitrogen-free Fahraeus solution, and cultivation was performed in a green house with a 16 h light and 8 h darkness cycle at 22°C and 20°C, respectively. Root nodules were harvested 28 or 50 days after *M. huakuii* 7653R inoculation.

### Extraction of total RNA and northern blotting

Total RNA was extracted from *M. huakuii* 7653R to detect the selected sRNAs by Northern blotting. Following exposure to stress conditions, cells were quickly centrifuged, resuspended in TRIzol Reagent (Roche, USA), and immediately frozen in liquid nitrogen and stored at –80°C. Plant nodules were resuspended in TRIzol Reagent and frozen in liquid nitrogen, then immediately subjected to grinding using a baked mortar and pestle to crush the nodule tissues. Isolation of total RNA and Northern blotting were conducted according to the manufacturer's protocols. Crude RNA extracts were purified with DNaseI to remove potential genomic DNA, and the resulting RNA was stored at –80°C. Northern hybridizations were performed with end-labeled [γ^−32P^]ATP DNA probes (Table S7). Hybridization signals were monitored using a radioisotope imaging system (Fujifilm, FLA 5100). The 5S rRNA was used as a control, and hybridization band intensities were relatively quantified with the Biorad Quantity One software.

### Extraction and purification of total RNA and deep sequencing of cDNAs

Total RNA was extracted using an RNeasy Mini Kit (Qiagen) following the manufacturer's instructions to carry out the RNA deep sequencing. The RNA integrity number (RIN) of the total RNA samples was checked using an Agilent Bioanalyzer 2100 (Agilent). Qualified total RNA was further purified using an RNeasy micro kit (QIAGEN) and RNase-Free DNase Set (QIAGEN) according to the manufacturer's instructions.

Total RNA, including small-sized RNAs from 28 dpi (days post-infection) nodules, 50 dpi nodules and free-living cells at OD_600_ = 0.8, was isolated and separated into small (< 100 nt) and long (> 100 nt) fractions using a miRNeasy Mini Kit (Qiagen) or a mirVana miRNA Isolation Kit (Ambion), respectively, according to the manufacturer's instructions. Total purified RNA was then treated with tobacco acid pyrophosphatase (TAP) (2.5 U/5 μg RNA in 50 μl for 2 h) to maintain the start regions of the transcription products. The quantity and quality of the processed RNA samples were assessed using of a NanoDrop ND-1000 spectrophotometer and Agilent 2100 Bioanalyzer. Qualified RNA was ligated to 5′- and 3′-RNA adapters by PCR, then the free adapters were removed and the remaining cDNA was purified. A Qubit® 2.0 Fluorometer and Agilent2100 instrument were used to monitor the abundance and size of cDNA, respectively. Solexa sequencing was performed using an Illumina HiSeq 2500. After that, all sequencing data were mapped by using the genomic sequence of *M. huakuii* 7653R to obtain the effective reads.

### Target prediction and the gene ontology (GO) analysis of candidate sRNAs

In order to figure out the potential function of the 16 new sRNA candidates identified in this work, we predicted the target genes for each of these sRNAs by using the webserver IntaRNA (http://rna.informatik.uni-freiburg.de/IntaRNA/Input.jsp). All of the encoded protein sequences of predicted target genes for each candidate sRNA were submitted to InterPro (http://www.ebi.ac.uk/interpro/sequence-search) for protein sequence analysis & classification, and the GO annotations (GO_ID) were extracted from the output. Further, the WEGO (Web Gene Ontology Annotation Plot) webserver (http://wego.genomics.org.cn/cgi-bin/wego/index.pl) was used to display the GO annotation results in histograms. The enriched GO terms of candidate small RNA target genes were summarized and demonstrated in a pie chart.

### Other tools and software

Searches for promoters in the *M. huakuii* 7653R genome were performed using Promoter 2.0 (http://www.cbs.dtu.dk/services/Promoter/) and BPROM 2.4.3.1111 (http://www.molquest.com/). Predictions of Rho-independent terminators were implemented with RNAMotif 3.0.7 (http://casegroup.rutgers.edu/casegr-sh-2.5.html), TransTermHP 2.09 (transterm.cbcb.umd.edu/), FindTerm 2.4.3.1111 (http://www.molquest.com/) and Erpin 5.4 (http://tagc.univ-mrs.fr/erpin/). Searches for known functional RNA sequences among the predicted sRNAs were conducted within the Rfam database using Rfam, version 12.0 (http://rfam.xfam.org/search) (Nawrocki et al., 2015). RNA secondary structures were analyzed using RNAfold 2.1.9 (http://rna.tbi.univie.ac.at/cgi-bin/RNAWebSuite/RNAfold.cgi). The target genes for the identified sRNAs were predicted using IntaRNA soft (http://rna.informatik.uni-freiburg.de/).

## Author contributions

XF and LY conceived and designed the experiments. ZW, ZJ and XF prepared RNA samples and conducted the analysis of the RNA-seq data. ZZ and HB predicted and identified the sRNAs in the *M. huakuii* 7653R genome. WX and MB performed the functional analysis of the target genes. The article was written and revised by XF, ZW, and LY.

### Conflict of interest statement

The authors declare that the research was conducted in the absence of any commercial or financial relationships that could be construed as a potential conflict of interest.
