# Supplementary material for: A Genome-Wide Prediction and Identification of Intergenic Small RNAs by Comparative Analysis in Mesorhizobium huakuii 7653R
Source: Front Microbiol. 2017 Sep 8;8:1730. doi: 10.3389/fmicb.2017.01730 (PMC5596092; doi:10.3389/fmicb.2017.01730)
Supplement: Supplementary file 1 [file DataSheet1.doc]

**The genome-wide prediction and identification of small RNAs by comparative analysis and RNA-seq in *Mesorhizobium huakuii* 7653R**

**Authors:**

Fuli Xie, Wenlong Zhao, Xiao Wang, Jing Zhang, Baohai Hao, Zhengzheng Zou, Bing-Guang Ma, Youguo Li^*^

**Address:**

State Key Laboratory of Agricultural Microbiology, Huazhong Agricultural University, Wuhan 430070, People’s Republic of China

**Corresponding author:**

Youguo Li

Tel: (86) 27 8728-1685, Fax: (86) 27 8728-0670, Email: youguoli@mail.hzau.edu.cn

**Supplementary Tables**

**Supplementary Table S1. The numbers of Rho-independent terminators predicted by RNAMotif, TransTermHP, FindTerm and Erpin**

| Strain | RNAMotif | Erpin | TransTermHP | FindTerm |
| --- | --- | --- | --- | --- |
| *M.hukuii* 7653R  (pMu7653Ra/ pMu7653Rb) | 2225 (54/86) * | 774 (17/21) | 1084 (23/26) | 1435 (36/54) |
| *M. australicum* WSM2073 | 2127 | 621 | 939 | 1397 |
| *M. ciceri* WSM1271  ([pMESCI01](http://www.ncbi.nlm.nih.gov/nuccore/NC_014918.1) ) | 2186 (142) | 682 (16) | 1002 (57) | 1478 (78) |
| *M.loti* MAFF303099  (pMLa/pMLb) | 2447 (105/74) | 808 (20/14) | 1204 (35/30) | 1617 (57/37) |
| *M. opportunistum* WSM2075 | 2311 | 680 | 1082 | 1444 |

* chromosome (plasmid-1/plasmid-2)

**Supplementary Table S2. The numbers of promoters predicted by Promoter 2.0 and BPROM**

| Strain | Promoter 2.0 | BPROM |
| --- | --- | --- |
| *M.hukuii* 7653R  (pMu7653Ra/ pMu7653Rb) | 3865 (140/249) * | 8264 (324/518) |
| *M. australicum* WSM2073 | 4018 | 8523 |
| *M. ciceri* WSM1271  ([pMESCI01](http://www.ncbi.nlm.nih.gov/nuccore/NC_014918.1) ) | 3911 (307) | 8852 (681) |
| *M.loti* MAFF303099  (pMLa/pMLb) | 4653 (274/165) | 9794 (619/370) |
| *M. opportunistum* WSM2075 | 4375 | 9556 |

*chromosome(plasmid-1/plasmid-2)

**Supplementary Table S3. The sequence depth and effective values of short (≤100 nt) fractions**

| Sample Name | Raw reads | Clean reads | Mapped reads | Mapping ratio |
| --- | --- | --- | --- | --- |
| Free-living cells | 19198680 | 18353831 | 17251282 | 93.99% |
| 20-dpi nodules | 19520938 | 18252736 | 9025437 | 49.45% |
| 50-dpi nodules | 20785155 | 19715159 | 9597573 | 48.68% |

**Supplementary Table S4. The sequence depth and effective values of long (≥100 nt) fractions**

| Sample Name | Raw reads | Clean reads | Mapped reads | Mapping ratio |
| --- | --- | --- | --- | --- |
| Free-living cells | 91,115,156 | 77,555,004 | 76,997,275 | 99.28% |
| 20-dpi nodules | 97,302,941 | 95,350,719 | 29,638,720 | 31.08% |
| 50-dpi nodules | 113,662,013 | 111,369,628 | 29,624,346 | 26.60% |

**Supplementary Table S5. The differentiation and fold-changes of candidate sRNA expression levels under different growth conditions based on the *M. huakuii* 7653R RNA-seq data**

| Candidate | IGR_id | Length | Sequence start and end  (transcriptional direction) | FC_rpkm | MN_rpkm | SN_rpkm | MN/FC | SN/FC |
| --- | --- | --- | --- | --- | --- | --- | --- | --- |
| MH_s1 | IGR_Pa-3 | 62 | 2599-2660(-) | 1394.33 | 381.45 | 365.20 | 0.27 | 0.26 |
| MH_s1* | IGR_Pa-3 | 126 | 2602-2727(+) | 101.16 | 50.98 | 60.11 | 0.50 | 0.59 |
| MH_s2 | IGR_Pa-5 | 74 | 4833-4906(+) | 54.87 | 29.87 | 22.69 | 0.54 | 0.41 |
| MH_s3 | IGR_Pa-50 | 189 | 61074-61262(-) | 48.03 | 571.52 | 261.50 | 11.90 | 5.44 |
| MH_s4 | IGR_Pb-1 | 98 | 2418-2515(-) | 911.45 | 302.92 | 314.35 | 0.33 | 0.34 |
| MH_s4* | IGR_Pb-1 | 131 | 2344-2474(+) | 147.02 | 157.55 | 104.17 | 1.07 | 0.71 |
| MH_s5 | IGR_Pb-19 | 441 | 27704-28144(+) | 0 | 54.57 | 34.29 | NO | NO |
| MH_s7 | IGR_G-58 | 170 | 72492-72661(-) | 23.89 | 112.68 | 183.77 | 4.72 | 7.69 |
| MH_s8 | IGR_G-605 | 125 | 866182-866302(-) | 25.33 | 23.96 | 13.33 | 0.95 | 0.53 |
| MH_s10 | IGR_G-893 | 141 | 1235474-1235614(+) | 15.51 | 18.25 | 24.69 | 1.18 | 1.59 |
| MH_s11 | IGR_G-1069 | 165 | 1432008-1432172(+) | 65.36 | 218.72 | 310.62 | 3.35 | 4.75 |
| MH_s12 | IGR_G-1203 | 85 | 1559651-1559735(+) | 82.38 | 52.33 | 64.56 | 0.64 | 0.78 |
| MH_s13 | IGR_G-1629 | 366 | 2051956-2052321(+) | 132.73 | 45.88 | 0 | 0.35 | 0 |
| MH_s13* | IGR_G-1629 | 131 | 2052083-2052213(+) | 0 | 385.01 | 132.83 | NO | NO |
| MH_s14 | IGR_G-2003 | 260 | 2506941-2507200(+) | 40.40 | 0 | 15.54 | 0 | 0.38 |
| MH_s15 | IGR_G-2184 | 204 | 2726658-2726861(-) | 32.58 | 12.20 | 12.85 | 0.37 | 0.39 |
| MH_s16 | IGR_G-2240 | 123 | 2796947-2797069(-) | 191.21 | 30.07 | 15.98 | NO | NO |
| MH_s18 | IGR_G-2341 | 222 | 2931996-2932217(+) | 43.86 | 99.91 | 78.15 | 2.28 | 1.78 |
| MH_s19 | IGR_G-2430 | 90 | 3053513-3053602(-) | 163.27 | 72.76 | 70.51 | 0.45 | 0.43 |
| MH_s20 | IGR_G-2487 | 403 | 3136500-3136902(-) | 692.90 | 1039.98 | 1205.31 | 1.50 | 1.74 |
| MH_s22 | IGR_G-2770 | 186 | 3491690-3491875(+) | 11.22 | 0 | 0 | 0 | 0 |
| MH_s23 | IGR_G-3495 | 325 | 4347914-4348238(-) | 0 | 62.50 | 52.94 | NO | NO |
| MH_s24 | IGR_G-3535 | 71 | 4403128-4403198(-) | 0 | 0 | 20.03 | NO | NO |
| MH_s25 | IGR_G-3642 | 187 | 4532144-4532330(-) | 53.44 | 48.15 | 58.84 | 0.90 | 1.10 |
| MH_s25* | IGR_G-3642 | 268 | 4531972-4532239(-) | 19.77 | 20.70 | 23.41 | 1.05 | 1.18 |
| MH_s26 | IGR_G-3682 | 209 | 4579346-4579554(-) | 0 | 581.31 | 748.99 | NO | NO |
| MH_s27 | IGR_G-3753 | 83 | 4677314-4677396(+) | 0 | 33.50 | 22.18 | NO | NO |
| MH_s28 | IGR_G-3754 | 75 | 4681648-4681722(+) | 0 | 34.81 | 21.80 | NO | NO |
| MH_s29 | IGR_G-3840 | 181 | 4780323-4780503(+) | 147.77 | 160.50 | 139.01 | 1.09 | 0.94 |
| MH_s30 | IGR_G-4067 | 132 | 5045561-5045692(-) | 19.75 | 0 | 0 | 0 | 0 |
| MH_s31 | IGR_G-4090 | 104 | 5068057-5068160(-) | 52.90 | 0 | 50.66 | 0 | 0.96 |
| MH_s32 | IGR_G-4120 | 140 | 5092499-5092638(-) | 1244.63 | 1253.26 | 1299.15 | 1.01 | 1.04 |
| MH_s33 | IGR_G-4190 | 215 | 5178945-5179159(-) | 234.98 | 560.89 | 591.76 | 2.39 | 2.52 |
| MH_s34 | IGR_G-4348 | 100 | 5355042-5355141(-) | 0 | 383.92 | 287.88 | NO | NO |
| MH_s35 | IGR_G-4638 | 135 | 5723811-5723945(+) | 0 | 0 | 22.41 | NO | NO |
| MH_s36 | IGR_G-4677 | 73 | 5779015-5779087(-) | 53.52 | 29.13 | 29.56 | 0.54 | 0.55 |
| MH_s37 | IGR_G-4735 | 65 | 5845359-5845423(-) | 0 | 141.23 | 236.33 | NO | NO |
| MH_s39 | IGR_G-4878 | 250 | 6026852-6027101(+) | 27.60 | 0 | 13.54 | 0 | 0.49 |
| MH_s40 | IGR_G-5080 | 289 | 6301885-6302173(+) | 523.54 | 883.04 | 859.06 | 1.69 | 1.64 |

* Additional transcription was detected in the predicted region or the opposite strand.

FC represents free-living cells, MN represents mature nodules (28 dpi) and SN represents senescent (50 dpi) nodules. No means the gene expression in free-living cells is zero.

**Supplementary Table S6. The target genes and potential interactive regions of MH_s15 sRNA predicted by the IntaRNA software**

| Target gene | interactive regions | Function of target gene |
| --- | --- | --- |
| *MCHK_RS17255* | 124 -147 | transcriptional regulator |
| *MCHK_RS23385* | 95 - 137 | ribose ABC transporter permease |
| *MCHK_RS14890* | 4 - 26 | hypothetical protein |
| *MCHK_RS19705* | 84 - 149 | Protein Homology |
| *MCHK_RS07250* | 57 - 84 | TetR family transcriptional regulator |
| *MCHK_RS31120* | 29 - 55 | FAD-dependent oxidoreductase |
| *MCHK_RS15770* | 40 - 59 | GntR family transcriptional regulator |
| *MCHK_RS04695* | 128 - 149 | CopG family transcriptional regulator |
| *MCHK_RS28780* | 24 - 63 | translation initiation factor IF-3 |
| *MCHK_RS19610* | 66 - 108 | PadR family transcriptional regulator |

**Supplementary Table S7: The sRNA probes used for Northern blotting**

| Detected sRNAs | Sequence |
| --- | --- |
| MH_s3 (+) | CTTCTTTTTTGCAAACATGAGGC |
| MH_s3 (-) | GCCTCATGTTTGCAAAAAAGAAG |
| MH_s7 (+) | CAAATGCGATCTTTGATGGTTGCTATACC |
| MH_s7 (-) | GGTATAGCAACCATCAAAGATCGCATTTG |
| MH_s10 (+) | CGGGAAAAGAAAGCGCGTCGAAGAAAAGCC |
| MH_s10 (-) | GGCTTTTCTTCGACGCGCTTTCTTTTCCCG |
| MH_s11 (+) | TAGAGCACCGGCCTTCTAAGCCGATGGTCACAG |
| MH_s11 (-) | CTGTGACCATCGGCTTAGAAGGCCGGTGCTCTA |
| MH_s15(+) | ACACATCCGAACCCGGCTCGCATTCCCGCCCCGACCC |
| MH_s15 (-) | GGGTCGGGGCGGGAATGCGAGCCGGGTTCGGATGTGT |
| MH_s22 (+) | TGAACACTTCTGACTTGCAAATTGATTGAG |
| MH_s22 (-) | CTCAATCAATTTGCAAGTCAGAAGTGTTCA |
| MH_s25 (+) | AAAGTATTACTTGGGCTCTATCGGAAATTCACG |
| MH_s25 (-) | CGTGAATTTCCGATAGAGCCCAAGTAATACTTT |
| MH_s36 (+) | CCAACGGCCTTGTACATGCTAAGATCCTGTC |
| MH_s36 (-) | GACAGGATCTTAGCATGTACAAGGCCGTTGG |
| MH_s39 (+) | TAAGACGCCCGCCCCAGCCCTGGACGCATTCGAC |
| MH_s39 (-) | GTCGAATGCGTCCAGGGCTGGGGCGGGCGTCTTA |

**Supplementary Figures:**

**Supplementary Figure S1:**

**
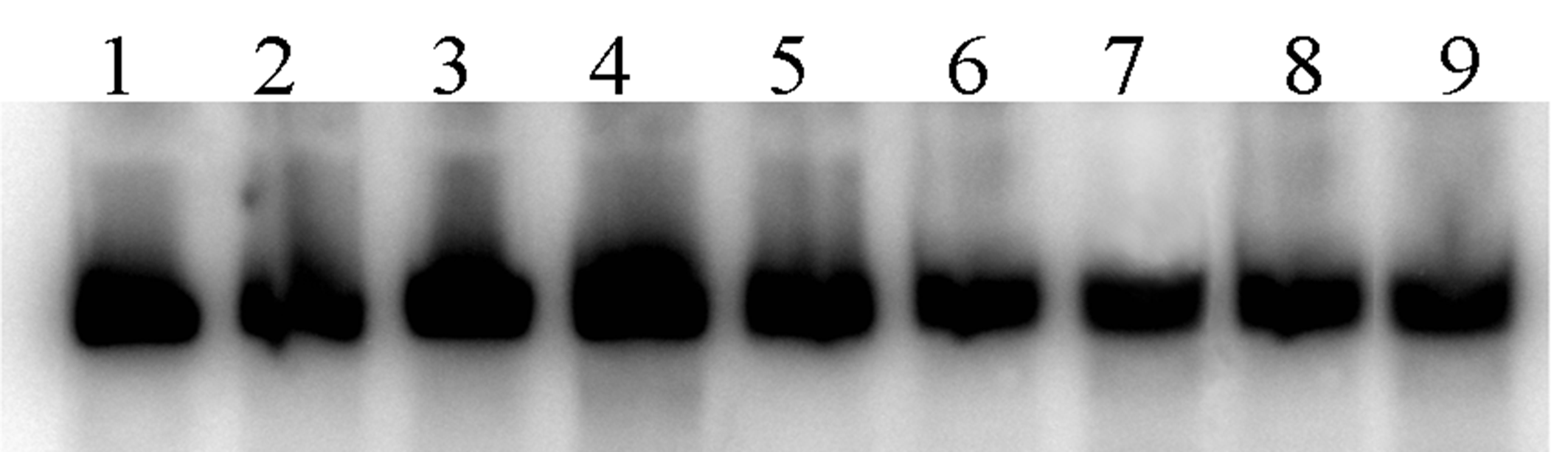
**

Figure S1: 5S RNA was used as the loading control in northern blotting.

Lanes 1–9 represent different tested conditions, as described in Figure 1.


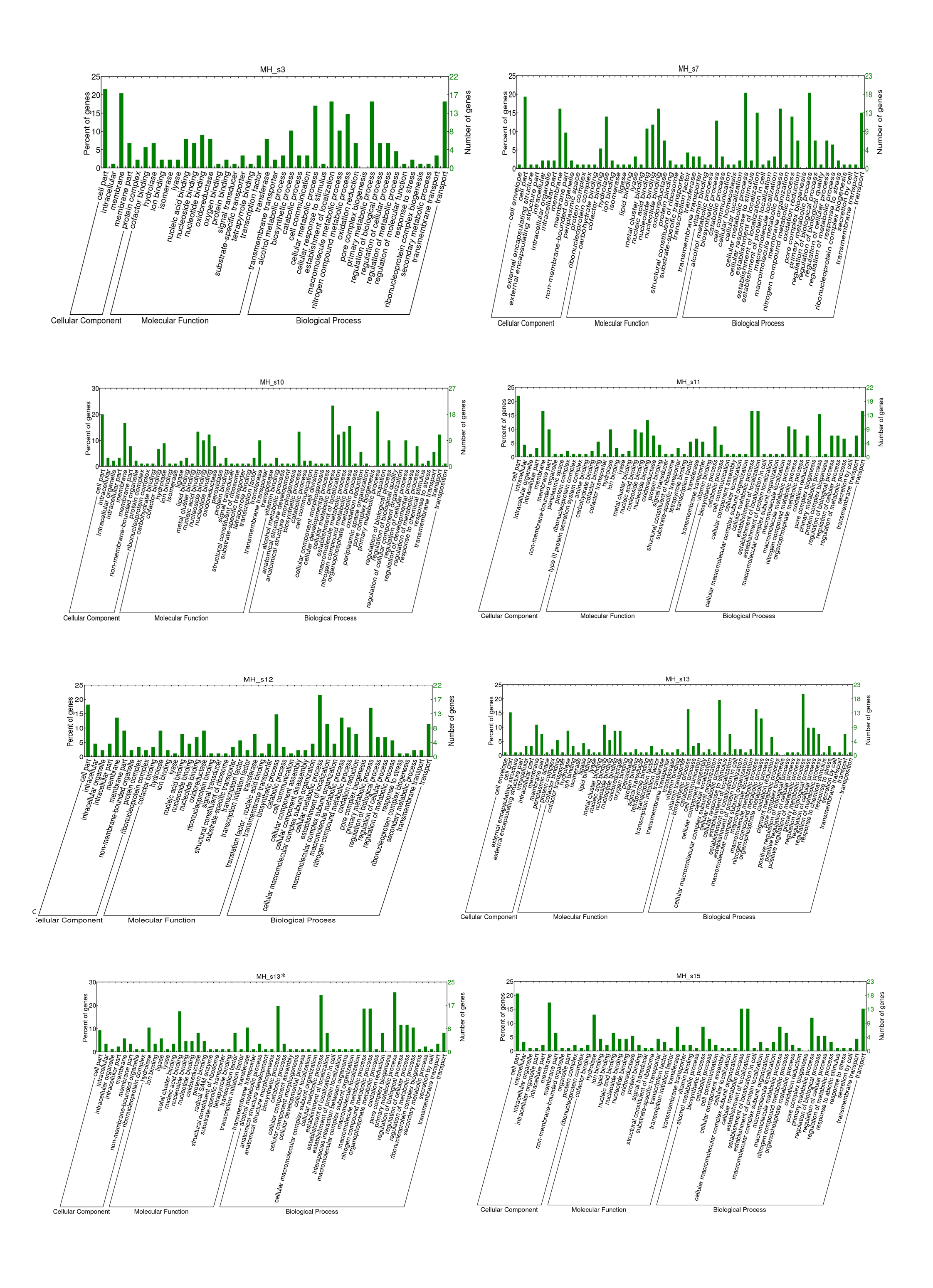
Figure S2: The WEGO functional categorization of candidate small RNA target genes

Figure S3:
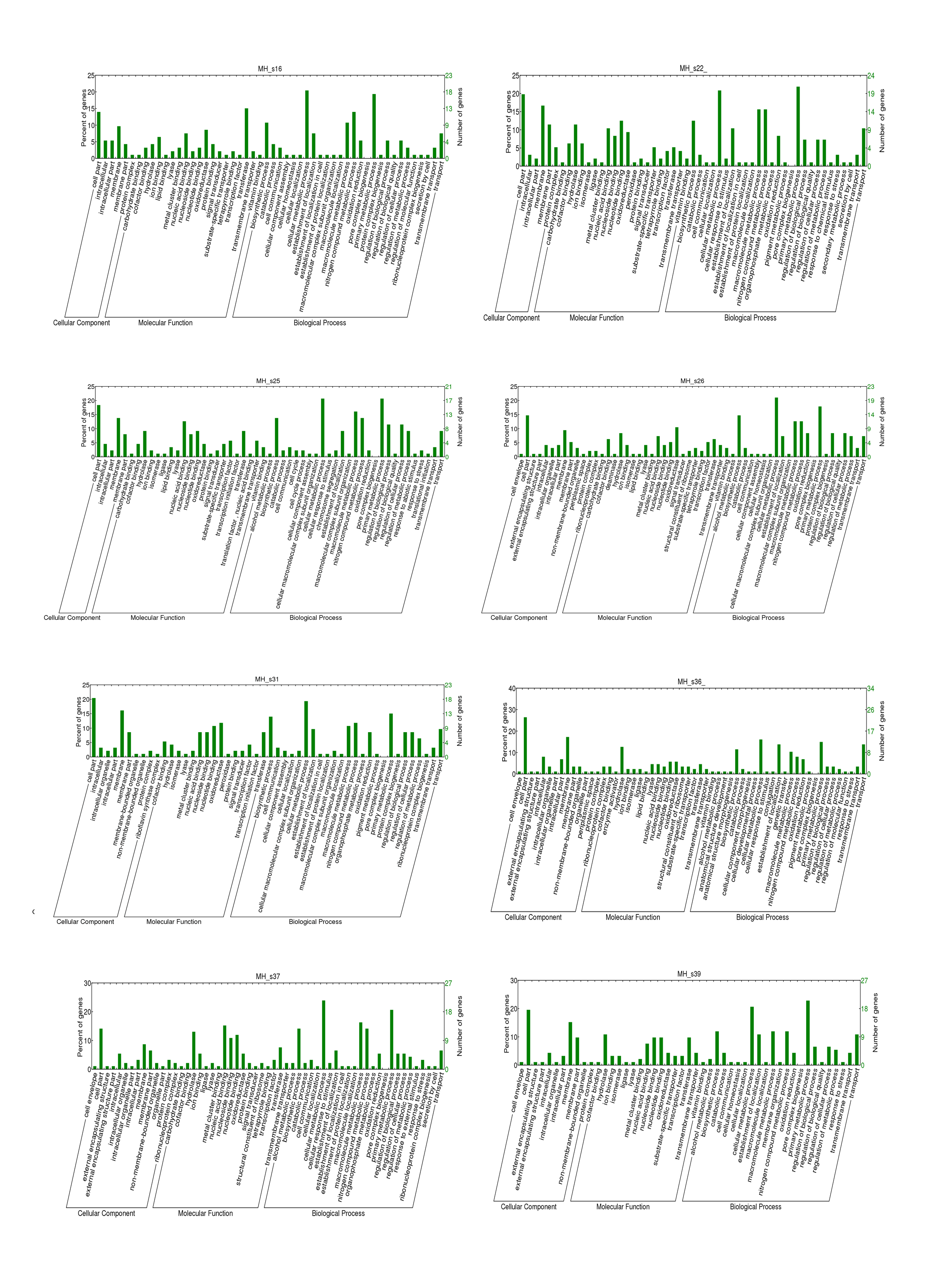
 The WEGO functional categorization of candidate small RNA target genes
