# Supplementary material for: A Genome-Wide Prediction and Identification of Intergenic Small RNAs by Comparative Analysis in Mesorhizobium huakuii 7653R
Source: Front Microbiol. 2017 Sep 8;8:1730. doi: 10.3389/fmicb.2017.01730 (PMC5596092; doi:10.3389/fmicb.2017.01730)
Supplement: Supplementary file 3 [file DataSheet3.doc]

| **Table S8.** Complete list of enriched GO terms of target genes in *Mesorhizobium huakuii* 7653R | | | | |
| --- | --- | --- | --- | --- |
| **Category** | **GO_ID** | **Description** | Genes Involved | |
| Biological Process | GO:0000160 | phosphorelay signal transduction system | MCHK_RS16805  MCHK_RS00370  MCHK_RS01000  MCHK_RS05730  MCHK_RS25760  MCHK_RS23995  MCHK_RS21725  MCHK_RS20495  MCHK_RS00980 | MCHK_RS23255  MCHK_RS04740  MCHK_RS05435  MCHK_RS29680  MCHK_RS05435  MCHK_RS17305  MCHK_RS16030  MCHK_RS32590 |
| Biological Process | GO:0000271 | polysaccharide biosynthetic process | MCHK_RS27305 |  |
| Biological Process | GO:0000287 | magnesium ion binding | MCHK_RS20295  MCHK_RS09725 | MCHK_RS24985 |
| Biological Process | GO:0000413 | protein peptidyl-prolyl isomerization | MCHK_RS11470 |  |
| Biological Process | GO:0000902 | cell morphogenesis | MCHK_RS16570 |  |
| Biological Process | GO:0002949 | tRNA threonylcarbamoyladenosine modification | MCHK_RS28880 | MCHK_RS30705 |
| Biological Process | GO:0003333 | amino acid transmembrane transport | MCHK_RS31755  MCHK_RS18490 | MCHK_RS24440 |
| Biological Process | GO:0005992 | trehalose biosynthetic process | MCHK_RS11295 | MCHK_RS11285 |
| Biological Process | GO:0006015 | 5-phosphoribose 1-diphosphate biosynthetic process | MCHK_RS28545 |  |
| Biological Process | GO:0006044 | N-acetylglucosamine metabolic process | MCHK_RS27420 |  |
| Biological Process | GO:0006071 | glycerol metabolic process | MCHK_RS12590 | MCHK_RS29790 |
| Biological Process | GO:0006094 | gluconeogenesis | MCHK_RS28920 | MCHK_RS12590 |
| Biological Process | GO:0006096 | glycolytic process | MCHK_RS03815 | MCHK_RS12425 |
| Biological Process | GO:0006099 | tricarboxylic acid cycle | MCHK_RS24350  MCHK_RS25795 | MCHK_RS13930 |
| Biological Process | GO:0006139 | nucleobase-containing compound metabolic process | MCHK_RS11735 |  |
| Biological Process | GO:0006168 | adenine salvage | MCHK_RS20160 |  |
| Biological Process | GO:0006189 | 'de novo' IMP biosynthetic process | MCHK_RS24245  MCHK_RS24395 | MCHK_RS06500 |
| Biological Process | GO:0006221 | pyrimidine nucleotide biosynthetic process | MCHK_RS11110  MCHK_RS05695 | MCHK_RS11265 |
| Biological Process | GO:0006259 | DNA metabolic process | MCHK_RS07975 |  |
| Biological Process | GO:0006260 | DNA replication | MCHK_RS06200  MCHK_RS11525 | MCHK_RS06080 |
| Biological Process | GO:0006265 | DNA topological change | MCHK_RS07975 | MCHK_RS12310 |
| Biological Process | GO:0006281 | DNA repair | MCHK_RS25705  MCHK_RS00060  MCHK_RS01550  MCHK_RS24605 | MCHK_RS21930  MCHK_RS16965  MCHK_RS19795  MCHK_RS12935 |
| Biological Process | GO:0006289 | nucleotide-excision repair | MCHK_RS11715 | MCHK_RS19795 |
| Biological Process | GO:0006298 | mismatch repair | MCHK_RS29945 |  |
| Biological Process | GO:0006310 | DNA recombination | MCHK_RS01550  MCHK_RS24605  MCHK_RS06540 | MCHK_RS09610  MCHK_RS13155 |
| Biological Process | GO:0006313 | transposition, DNA-mediated | MCHK_RS12300 | MCHK_RS30950 |
| Biological Process | GO:0006351 | transcription, DNA-templated | MCHK_RS09830 |  |
| Biological Process | GO:0006352 | DNA-templated transcription, initiation | MCHK_RS29015 |  |
| Biological Process | GO:0006355 | regulation of transcription, DNA-templated | MCHK_RS00385  MCHK_RS01625  MCHK_RS01940  MCHK_RS02590  MCHK_RS03230  MCHK_RS03805  MCHK_RS05190  MCHK_RS05295  MCHK_RS05365  MCHK_RS05405  MCHK_RS05495  MCHK_RS05895  MCHK_RS05905  MCHK_RS05965  MCHK_RS06925  MCHK_RS07115  MCHK_RS07285  MCHK_RS07290  MCHK_RS07570  MCHK_RS08040  MCHK_RS08900  MCHK_RS09125  MCHK_RS10290  MCHK_RS10300  MCHK_RS13080  MCHK_RS13295  MCHK_RS14585  MCHK_RS15555  MCHK_RS15580  MCHK_RS15980  MCHK_RS16155  MCHK_RS16300  MCHK_RS16660  MCHK_RS17300  MCHK_RS17350  MCHK_RS17475 | MCHK_RS18365  MCHK_RS18900  MCHK_RS18935  MCHK_RS19055  MCHK_RS19250  MCHK_RS19510  MCHK_RS19675  MCHK_RS20525  MCHK_RS20775  MCHK_RS22290  MCHK_RS23165  MCHK_RS24590  MCHK_RS26640  MCHK_RS27460  MCHK_RS27805  MCHK_RS29960  MCHK_RS30525  MCHK_RS32205  MCHK_RS32905  MCHK_RS33020  MCHK_RS33215  MCHK_RS00370  MCHK_RS05435  MCHK_RS05730  MCHK_RS16030  MCHK_RS21725  MCHK_RS23255  MCHK_RS25760  MCHK_RS32590  MCHK_RS21930  MCHK_RS18700  MCHK_RS23945  MCHK_RS24215  MCHK_RS27785  MCHK_RS29015 |
| Biological Process | GO:0006364 | rRNA processing | MCHK_RS11735 | MCHK_RS30810 |
| Biological Process | GO:0006396 | RNA processing | MCHK_RS30800  MCHK_RS22055 | MCHK_RS05735 |
| Biological Process | GO:0006400 | tRNA modification | MCHK_RS10540 |  |
| Biological Process | GO:0006402 | mRNA catabolic process | MCHK_RS20910 | MCHK_RS30800 |
| Biological Process | GO:0006412 | translation | MCHK_RS08475  MCHK_RS20030  MCHK_RS09965  MCHK_RS09930  MCHK_RS00020  MCHK_RS09895 | MCHK_RS09885  MCHK_RS24720  MCHK_RS11930  MCHK_RS09890  MCHK_RS09940  MCHK_RS09970 |
| Biological Process | GO:0006413 | translational initiation | MCHK_RS28765 |  |
| Biological Process | GO:0006415 | translational termination | MCHK_RS30040 |  |
| Biological Process | GO:0006418 | tRNA aminoacylation for protein translation | MCHK_RS00305  MCHK_RS12960  MCHK_RS25105 | MCHK_RS14005  MCHK_RS11105 |
| Biological Process | GO:0006450 | regulation of translational fidelity | MCHK_RS11245 |  |
| Biological Process | GO:0006457 | protein folding | MCHK_RS11470  MCHK_RS07250  MCHK_RS28170 | MCHK_RS27385  MCHK_RS16770 |
| Biological Process | GO:0006464 | cellular protein modification process | MCHK_RS12860 |  |
| Biological Process | GO:0006470 | protein dephosphorylation | MCHK_RS26255 |  |
| Biological Process | GO:0006474 | N-terminal protein amino acid acetylation | MCHK_RS30725 |  |
| Biological Process | GO:0006508 | proteolysis | MCHK_RS02125  MCHK_RS04265  MCHK_RS04375  MCHK_RS04635  MCHK_RS04755  MCHK_RS05280  MCHK_RS09485 | MCHK_RS10780  MCHK_RS11230  MCHK_RS20445  MCHK_RS21670  MCHK_RS22235  MCHK_RS29775 |
| Biological Process | GO:0006520 | cellular amino acid metabolic process | MCHK_RS26985 |  |
| Biological Process | GO:0006526 | arginine biosynthetic process | MCHK_RS22850 |  |
| Biological Process | GO:0006542 | glutamine biosynthetic process | MCHK_RS03955 | MCHK_RS29190 |
| Biological Process | GO:0006545 | glycine biosynthetic process | MCHK_RS14640 |  |
| Biological Process | GO:0006559 | L-phenylalanine catabolic process | MCHK_RS07715 |  |
| Biological Process | GO:0006596 | polyamine biosynthetic process | MCHK_RS21050 |  |
| Biological Process | GO:0008152 | metabolic process | MCHK_RS00325  MCHK_RS01270  MCHK_RS01310  MCHK_RS01815  MCHK_RS02005  MCHK_RS03375  MCHK_RS03910  MCHK_RS04200  MCHK_RS04580  MCHK_RS04775  MCHK_RS05175  MCHK_RS06405  MCHK_RS07670  MCHK_RS09060  MCHK_RS10375  MCHK_RS10995  MCHK_RS11995  MCHK_RS12080  MCHK_RS12200  MCHK_RS13320  MCHK_RS14670  MCHK_RS16205  MCHK_RS18025  MCHK_RS18215  MCHK_RS18780  MCHK_RS20410  MCHK_RS21180  MCHK_RS21415  MCHK_RS21475  MCHK_RS22815  MCHK_RS23220  MCHK_RS23545  MCHK_RS23805 | MCHK_RS24875  MCHK_RS25465  MCHK_RS27405  MCHK_RS27510  MCHK_RS28380  MCHK_RS28400  MCHK_RS28530  MCHK_RS28835  MCHK_RS28935  MCHK_RS29365  MCHK_RS30365  MCHK_RS30820  MCHK_RS32625  MCHK_RS32680  MCHK_RS33025  MCHK_RS03815  MCHK_RS13930  MCHK_RS12815  MCHK_RS09560  MCHK_RS22130  MCHK_RS13295  MCHK_RS22130  MCHK_RS07470  MCHK_RS07925  MCHK_RS24955  MCHK_RS22815  MCHK_RS23220  MCHK_RS28380  MCHK_RS28935  MCHK_RS21850  MCHK_RS14670  MCHK_RS22055 |
| Biological Process | GO:0006614 | SRP-dependent cotranslational protein targeting to membrane | MCHK_RS25990 |  |
| Biological Process | GO:0006629 | lipid metabolic process | MCHK_RS05795  MCHK_RS10180 | MCHK_RS12815  MCHK_RS08405 |
| Biological Process | GO:0006631 | fatty acid metabolic process | MCHK_RS14085 | MCHK_RS01970 |
| Biological Process | GO:0006633 | fatty acid biosynthetic process | MCHK_RS09425 | MCHK_RS12780 |
| Biological Process | GO:0006725 | cellular aromatic compound metabolic process | MCHK_RS03510 |  |
| Biological Process | GO:0006741 | NADP biosynthetic process | MCHK_RS09560 |  |
| Biological Process | GO:0006744 | ubiquinone biosynthetic process | MCHK_RS10570  MCHK_RS22775 |  |
| Biological Process | GO:0006754 | ATP biosynthetic process | MCHK_RS32065 |  |
| Biological Process | GO:0006777 | Mo-molybdopterin cofactor biosynthetic process | MCHK_RS06285 |  |
| Biological Process | GO:0006779 | porphyrin-containing compound biosynthetic process | MCHK_RS22130 |  |
| Biological Process | GO:0006793 | phosphorus metabolic process | MCHK_RS00245 |  |
| Biological Process | GO:0006807 | nitrogen compound metabolic process | MCHK_RS03955  MCHK_RS29190  MCHK_RS08350  MCHK_RS29950 | MCHK_RS11850  MCHK_RS28375  MCHK_RS14530 |
| Biological Process | GO:0006810 | transport | MCHK_RS00300  MCHK_RS00405  MCHK_RS00605  MCHK_RS00840  MCHK_RS01020  MCHK_RS01425  MCHK_RS01445  MCHK_RS03305  MCHK_RS03355  MCHK_RS03645  MCHK_RS03650  MCHK_RS03715  MCHK_RS03755  MCHK_RS04185  MCHK_RS04655  MCHK_RS05090  MCHK_RS05210  MCHK_RS05410  MCHK_RS05680  MCHK_RS05870  MCHK_RS06205  MCHK_RS07105  MCHK_RS08325  MCHK_RS08520  MCHK_RS09590  MCHK_RS11975  MCHK_RS14725  MCHK_RS15305  MCHK_RS15310  MCHK_RS15440  MCHK_RS15485  MCHK_RS15590  MCHK_RS15685  MCHK_RS15695  MCHK_RS15775  MCHK_RS16470 | MCHK_RS17125  MCHK_RS17130  MCHK_RS17490  MCHK_RS17490  MCHK_RS17665  MCHK_RS18035  MCHK_RS18055  MCHK_RS19340  MCHK_RS20755  MCHK_RS20935  MCHK_RS22410  MCHK_RS22505  MCHK_RS22690  MCHK_RS23500  MCHK_RS23610  MCHK_RS24115  MCHK_RS24195  MCHK_RS24450  MCHK_RS24745  MCHK_RS24745  MCHK_RS24745  MCHK_RS25500  MCHK_RS26710  MCHK_RS27490  MCHK_RS27515  MCHK_RS27695  MCHK_RS27735  MCHK_RS27750  MCHK_RS28290  MCHK_RS28965  MCHK_RS28975  MCHK_RS30405  MCHK_RS30640  MCHK_RS30650  MCHK_RS27305  MCHK_RS11230 |
| Biological Process | GO:0006812 | cation transport | MCHK_RS29930  MCHK_RS01700 | MCHK_RS11680  MCHK_RS21090 |
| Biological Process | GO:0006813 | potassium ion transport | MCHK_RS21620 | MCHK_RS11680 |
| Biological Process | GO:0006821 | chloride transport | MCHK_RS26135 |  |
| Biological Process | GO:0006859 | extracellular carbohydrate transport | MCHK_RS29750 |  |
| Biological Process | GO:0006865 | amino acid transport | MCHK_RS05630  MCHK_RS28520  MCHK_RS10830  MCHK_RS14560 | MCHK_RS26160  MCHK_RS24830  MCHK_RS18490 |
| Biological Process | GO:0006950 | response to stress | MCHK_RS24060  MCHK_RS17905 | MCHK_RS16770 |
| Biological Process | GO:0006979 | response to oxidative stress | MCHK_RS02445 | MCHK_RS21030 |
| Biological Process | GO:0007165 | signal transduction | MCHK_RS06445  MCHK_RS26340  MCHK_RS29855  MCHK_RS02120 | MCHK_RS26490  MCHK_RS17825  MCHK_RS10290 |
| Biological Process | GO:0008360 | regulation of cell shape | MCHK_RS15055 |  |
| Biological Process | GO:0008652 | cellular amino acid biosynthetic process | MCHK_RS22750 |  |
| Biological Process | GO:0008654 | phospholipid biosynthetic process | MCHK_RS11270 | MCHK_RS18745 |
| Biological Process | GO:0008033 | tRNA processing | MCHK_RS25895 | MCHK_RS27120 |
| Biological Process | GO:0015074 | DNA integration | MCHK_RS13180  MCHK_RS33265  MCHK_RS06540 | MCHK_RS09610  MCHK_RS13155 |
| Biological Process | GO:0015762 | rhamnose transport | MCHK_RS00500 |  |
| Biological Process | GO:0015774 | polysaccharide transport | MCHK_RS08415 |  |
| Biological Process | GO:0015833 | peptide transport | MCHK_RS30555 |  |
| Biological Process Biological Process | GO:0015846 | polyamine transport | MCHK_RS16815  MCHK_RS17125 | MCHK_RS20935 |
| Biological Process | GO:0015878 | biotin transport | MCHK_RS05005 |  |
| Biological Process | GO:0015886 | heme transport | MCHK_RS07765 |  |
| Biological Process | GO:0015940 | pantothenate biosynthetic process | MCHK_RS33230 |  |
| Biological Process | GO:0015969 | guanosine tetraphosphate metabolic process | MCHK_RS05690 |  |
| Biological Process | GO:0015992 | proton transport | MCHK_RS08180 | MCHK_RS32065 |
| Biological Process | GO:0015995 | chlorophyll biosynthetic process | MCHK_RS27765 |  |
| Molecular Function | GO:0016301 | kinase activity | MCHK_RS04980  MCHK_RS33025  MCHK_RS18780 | MCHK_RS23805  MCHK_RS15530 |
| Biological Process | GO:0016311 | dephosphorylation | MCHK_RS13850 |  |
| Biological Process | GO:0017003 | protein-heme linkage | MCHK_RS05555 |  |
| Biological Process | GO:0017004 | cytochrome complex assembly | MCHK_RS26030  MCHK_RS07765 | MCHK_RS05555 |
| Biological Process | GO:0019277 | UDP-N-acetylgalactosamine biosynthetic process | MCHK_RS03670 | MCHK_RS01740 |
| Biological Process | GO:0019299 | rhamnose metabolic process | MCHK_RS00480 |  |
| Biological Process | GO:0019475 | L-lysine catabolic process to acetate | MCHK_RS00030 | MCHK_RS09025 |
| Biological Process | GO:0019538 | protein metabolic process | MCHK_RS18135 |  |
| Biological Process | GO:0019700 | organic phosphonate catabolic process | MCHK_RS22375 |  |
| Biological Process | GO:0019825 | oxygen binding | MCHK_RS09855 |  |
| Biological Process | GO:0022900 | electron transport chain | MCHK_RS04720  MCHK_RS12785 | MCHK_RS04655 |
| Biological Process | GO:0030163 | protein catabolic process | MCHK_RS09420 |  |
| Biological Process | GO:0031167 | rRNA methylation | MCHK_RS07470 |  |
| Biological Process | GO:0034194 | D-galactonate catabolic process | MCHK_RS27325 |  |
| Biological Process | GO:0035444 | nickel cation transmembrane transport | MCHK_RS21080 |  |
| Biological Process | GO:0042128 | nitrate assimilation | MCHK_RS12070 |  |
| Biological Process | GO:0042773 | ATP synthesis coupled electron transport | MCHK_RS14025 |  |
| Biological Process | GO:0042953 | lipoprotein transport | MCHK_RS12390 | MCHK_RS13985 |
| Biological Process | GO:0045454 | cell redox homeostasis | MCHK_RS08840  MCHK_RS31370 | MCHK_RS26030 |
| Biological Process | GO:0046294 | formaldehyde catabolic process | MCHK_RS02970 | MCHK_RS12080 |
| Biological Process | GO:0046417 | chorismate metabolic process | MCHK_RS26200 |  |
| Biological Process | GO:0046653 | tetrahydrofolate metabolic process | MCHK_RS21765  MCHK_RS02875 | MCHK_RS04000 |
| Biological Process | GO:0046854 | phosphatidylinositol phosphorylation | MCHK_RS24670 | MCHK_RS21280 |
| Biological Process | GO:0048034 | heme O biosynthetic process | MCHK_RS12755 |  |
| Molecular Function | GO:0048037 | cofactor binding | MCHK_RS12395  MCHK_RS05145  MCHK_RS26005 | MCHK_RS15805  MCHK_RS12185 |
| Biological Process | GO:0051205 | protein insertion into membrane | MCHK_RS27680 |  |
| Biological Process | GO:0051304 | chromosome separation | MCHK_RS31765 | MCHK_RS12895 |
| Biological Process | GO:0009058 | biosynthetic process | MCHK_RS25060  MCHK_RS00455  MCHK_RS13780  MCHK_RS21155  MCHK_RS29745  MCHK_RS28870 | MCHK_RS08900  MCHK_RS19055  MCHK_RS32065  MCHK_RS15685  MCHK_RS01815  MCHK_RS15055 |
| Biological Process | GO:0009073 | aromatic amino acid family biosynthetic process | MCHK_RS23285 |  |
| Biological Process | GO:0009088 | threonine biosynthetic process | MCHK_RS04715 |  |
| Biological Process | GO:0009089 | lysine biosynthetic process via diaminopimelate | MCHK_RS23035 | MCHK_RS22750 |
| Biological Process | GO:0009107 | lipoate biosynthetic process | MCHK_RS10270 |  |
| Biological Process | GO:0009116 | nucleoside metabolic process | MCHK_RS12430  MCHK_RS02470 | MCHK_RS20160  MCHK_RS05695 |
| Biological Process | GO:0009132 | nucleoside diphosphate metabolic process | MCHK_RS20295 |  |
| Biological Process | GO:0009168 | purine ribonucleoside monophosphate biosynthetic process | MCHK_RS16120 |  |
| Biological Process | GO:0009228 | thiamine biosynthetic process | MCHK_RS16000 | MCHK_RS26425 |
| Biological Process | GO:0009231 | riboflavin biosynthetic process | MCHK_RS16280 | MCHK_RS03850 |
| Biological Process | GO:0009236 | cobalamin biosynthetic process | MCHK_RS14170  MCHK_RS13855  MCHK_RS22130 | MCHK_RS14670 |
| Biological Process | GO:0009245 | lipid A biosynthetic process | MCHK_RS08730 | MCHK_RS15015 |
| Biological Process | GO:0009253 | peptidoglycan catabolic process | MCHK_RS15180 |  |
| Biological Process | GO:0009291 | unidirectional conjugation | MCHK_RS31005 |  |
| Biological Process | GO:0009306 | protein secretion | MCHK_RS32085  MCHK_RS20735  MCHK_RS19815 | MCHK_RS19655  MCHK_RS32225  MCHK_RS07070 |
| Biological Process | GO:0009401 | phosphoenolpyruvate-dependent sugar phosphotransferase system | MCHK_RS23450 |  |
| Biological Process | GO:0009405 | pathogenesis | MCHK_RS11360 |  |
| Biological Process | GO:0009435 | NAD biosynthetic process | MCHK_RS04340  MCHK_RS11850 | MCHK_RS25060 |
| Biological Process | GO:0009436 | glyoxylate catabolic process | MCHK_RS09725 |  |
| Biological Process | GO:0009443 | pyridoxal 5'-phosphate salvage | MCHK_RS25315 |  |
| Biological Process | GO:0009448 | gamma-aminobutyric acid metabolic process | MCHK_RS30740 |  |
| Biological Process | GO:0052547 | regulation of peptidase activity | MCHK_RS14655 | MCHK_RS16485 |
| Biological Process | GO:0055070 | copper ion homeostasis | MCHK_RS12595 |  |
| Biological Process | GO:0055085 | transmembrane transport | MCHK_RS02075  MCHK_RS03840  MCHK_RS04060  MCHK_RS04325  MCHK_RS06140  MCHK_RS07705  MCHK_RS13495  MCHK_RS13505  MCHK_RS13885  MCHK_RS15560  MCHK_RS17100  MCHK_RS17275  MCHK_RS17810  MCHK_RS22000  MCHK_RS22860 | MCHK_RS24040  MCHK_RS24970  MCHK_RS25470  MCHK_RS26800  MCHK_RS30430  MCHK_RS30560  MCHK_RS15695  MCHK_RS17130  MCHK_RS27695  MCHK_RS27750  MCHK_RS21090  MCHK_RS26135  MCHK_RS19655  MCHK_RS11230  MCHK_RS11680 |
| Biological Process | GO:0055114 | oxidation-reduction process | MCHK_RS00975  MCHK_RS01215  MCHK_RS01275  MCHK_RS01500  MCHK_RS02150  MCHK_RS03055  MCHK_RS03310  MCHK_RS03920  MCHK_RS04175  MCHK_RS07080  MCHK_RS07220  MCHK_RS07615  MCHK_RS08050  MCHK_RS09205  MCHK_RS10330  MCHK_RS11605  MCHK_RS11745  MCHK_RS13475  MCHK_RS14075  MCHK_RS15385  MCHK_RS15570  MCHK_RS15605  MCHK_RS15845  MCHK_RS16210  MCHK_RS16495  MCHK_RS16865  MCHK_RS17290  MCHK_RS17325  MCHK_RS17545  MCHK_RS17900  MCHK_RS19290  MCHK_RS19700  MCHK_RS20080  MCHK_RS21205  MCHK_RS22175  MCHK_RS24405  MCHK_RS26860 | MCHK_RS27745  MCHK_RS27965  MCHK_RS28080  MCHK_RS28600  MCHK_RS29450  MCHK_RS29820  MCHK_RS30230  MCHK_RS30530  MCHK_RS32355  MCHK_RS32395  MCHK_RS33615  MCHK_RS13295  MCHK_RS04655  MCHK_RS25640  MCHK_RS03510  MCHK_RS07715  MCHK_RS13320  MCHK_RS14640  MCHK_RS22130  MCHK_RS01970  MCHK_RS14085  MCHK_RS10570  MCHK_RS02445  MCHK_RS21030  MCHK_RS01270  MCHK_RS18025  MCHK_RS21415  MCHK_RS21475  MCHK_RS28530  MCHK_RS29365  MCHK_RS30365  MCHK_RS33110  MCHK_RS16280  MCHK_RS27765  MCHK_RS14025  MCHK_RS04000  MCHK_RS21765 |
| Biological Process | GO:0071805 | potassium ion transmembrane transport | MCHK_RS02780 |  |
| Biological Process | GO:0071973 | bacterial-type flagellum-dependent cell motility | MCHK_RS20830 |  |
| Biological Process | GO:1901135 | carbohydrate derivative metabolic process | MCHK_RS08490  MCHK_RS27430 | MCHK_RS19525  MCHK_RS16300 |
| Biological Process | GO:0005978 | glycogen biosynthetic process | MCHK_RS05015 |  |
| Biological Process | GO:0006072 | glycerol-3-phosphate metabolic process | MCHK_RS25640 |  |
| Biological Process | GO:0006109 | regulation of carbohydrate metabolic process | MCHK_RS29680 |  |
| Biological Process | GO:0006424 | glutamyl-tRNA aminoacylation | MCHK_RS11105 |  |
| Biological Process | GO:0006429 | leucyl-tRNA aminoacylation | MCHK_RS25105 |  |
| Biological Process | GO:0006430 | lysyl-tRNA aminoacylation | MCHK_RS00305 |  |
| Biological Process | GO:0006433 | prolyl-tRNA aminoacylation | MCHK_RS14005 |  |
| Biological Process | GO:0006438 | valyl-tRNA aminoacylation | MCHK_RS12960 |  |
| Biological Process | GO:0006461 | protein complex assembly | MCHK_RS28170 |  |
| Biological Process | GO:0006527 | arginine catabolic process | MCHK_RS18490 |  |
| Biological Process | GO:0006561 | proline biosynthetic process | MCHK_RS13295 |  |
| Biological Process | GO:0006570 | tyrosine metabolic process | MCHK_RS07715 |  |
| Biological Process | GO:0008616 | queuosine biosynthetic process | MCHK_RS27120 |  |
| Biological Process | GO:0009060 | aerobic respiration | MCHK_RS04655 |  |
| Biological Process | GO:0009103 | lipopolysaccharide biosynthetic process | MCHK_RS29750 |  |
| Biological Process | GO:0009165 | nucleotide biosynthetic process | MCHK_RS14640 |  |
| Biological Process | GO:0009396 | folic acid-containing compound biosynthetic process | MCHK_RS21155  MCHK_RS28870 | MCHK_RS15055 |
| Biological Process | GO:0009450 | gamma-aminobutyric acid catabolic process | MCHK_RS13320 |  |
| Biological Process | GO:0015031 | protein transport | MCHK_RS07070  MCHK_RS19815 | MCHK_RS20735  MCHK_RS32085 |
| Biological Process | GO:0016075 | rRNA catabolic process | MCHK_RS05735 |  |
| Biological Process | GO:0016114 | terpenoid biosynthetic process | MCHK_RS04580 |  |
| Biological Process | GO:0016310 | phosphorylation | MCHK_RS02120  MCHK_RS06445  MCHK_RS26340 | MCHK_RS26490  MCHK_RS29855  MCHK_RS10290 |
| Biological Process | GO:0016998 | cell wall macromolecule catabolic process | MCHK_RS15180 |  |
| Biological Process | GO:0018339 | peptidyl-L-beta-methylthioaspartic acid biosynthetic process from peptidyl-aspartic acid | MCHK_RS10540 |  |
| Biological Process | GO:0019428 | allantoin biosynthetic process | MCHK_RS29070 |  |
| Biological Process | GO:0019439 | aromatic compound catabolic process | MCHK_RS03510 |  |
| Biological Process | GO:0030001 | metal ion transport | MCHK_RS01700 |  |
| Biological Process | GO:0030488 | tRNA methylation | MCHK_RS26130 |  |
| Biological Process | GO:0032259 | methylation | MCHK_RS07925 | MCHK_RS07470 |
| Biological Process | GO:0045893 | positive regulation of transcription, DNA-templated | MCHK_RS29960 |  |
| Biological Process | GO:0046855 | inositol phosphate dephosphorylation | MCHK_RS24670 |  |
| Biological Process | GO:0051156 | glucose 6-phosphate metabolic process | MCHK_RS12425 |  |
| Biological Process | GO:0051726 | regulation of cell cycle | MCHK_RS16570 |  |
| Biological Process | GO:1902209 | negative regulation of bacterial-type flagellum assembly | MCHK_RS20910 |  |
| Biological Process | GO:0009408 | response to heat | MCHK_RS23945 |  |
| Biological Process | GO:0009432 | SOS response | MCHK_RS19795 |  |
| Biological Process | GO:0018307 | enzyme active site formation | MCHK_RS28170 |  |
| Biological Process | GO:0019674 | NAD metabolic process | MCHK_RS09560 |  |
| Biological Process | GO:0043039 | tRNA aminoacylation | MCHK_RS11105 |  |
| Biological Process | GO:0044780 | bacterial-type flagellum assembly | MCHK_RS20735 |  |
| Biological Process | GO:0019856 | pyrimidine nucleobase biosynthetic process | MCHK_RS05695 |  |
| Biological Process | GO:0045226 | extracellular polysaccharide biosynthetic process | MCHK_RS29750 |  |
| Biological Process | GO:0046168 | glycerol-3-phosphate catabolic process | MCHK_RS25640 |  |
| Biological Process | GO:0070475 | rRNA base methylation | MCHK_RS26130 |  |
| Biological Process | GO:1901891 | regulation of cell septum assembly | MCHK_RS16570 |  |
| Biological Process | GO:0010133 | proline catabolic process to glutamate | MCHK_RS13295 |  |
| Biological Process | GO:0019354 | siroheme biosynthetic process | MCHK_RS22130 |  |
| Biological Process | GO:0019627 | urea metabolic process | MCHK_RS28170 |  |
| Biological Process | GO:0051301 | cell division | MCHK_RS15055 |  |
| Biological Process | GO:0046034 | ATP metabolic process | MCHK_RS32065 |  |
| Cellular Component | GO:0003735 | structural constituent of ribosome | MCHK_RS08475  MCHK_RS09885  MCHK_RS09890  MCHK_RS09930  MCHK_RS09940  MCHK_RS09965 | MCHK_RS09970  MCHK_RS11930  MCHK_RS20030  MCHK_RS24720  MCHK_RS00020  MCHK_RS09895 |
| Cellular Component | GO:0005737 | cytoplasm | MCHK_RS30565  MCHK_RS11105  MCHK_RS00480  MCHK_RS06500  MCHK_RS12780  MCHK_RS24955  MCHK_RS20160  MCHK_RS07250  MCHK_RS30040  MCHK_RS22765 | MCHK_RS23285  MCHK_RS11110  MCHK_RS28170  MCHK_RS14005  MCHK_RS26130  MCHK_RS10540  MCHK_RS00305  MCHK_RS12960  MCHK_RS15055  MCHK_RS32065 |
| Cellular Component | GO:0071555 | cell wall organization | MCHK_RS17305 |  |
| Cellular Component | GO:0005840 | ribosome | MCHK_RS08475  MCHK_RS09885  MCHK_RS09890  MCHK_RS09930  MCHK_RS09965  MCHK_RS11930 | MCHK_RS24720  MCHK_RS20030  MCHK_RS00020  MCHK_RS09970  MCHK_RS09895  MCHK_RS25885 |
| Cellular Component | GO:0005886 | plasma membrane | MCHK_RS25565  MCHK_RS26020  MCHK_RS23840  MCHK_RS28795  MCHK_RS21775 | MCHK_RS07070  MCHK_RS04655  MCHK_RS05005  MCHK_RS05555  MCHK_RS11270 |
| Cellular Component | GO:0009349 | riboflavin synthase complex | MCHK_RS03850 |  |
| Cellular Component | GO:0015934 | large ribosomal subunit | MCHK_RS09940 | MCHK_RS09895 |
| Cellular Component | GO:0030254 | protein secretion by the type III secretion system | MCHK_RS32065 |  |
| Cellular Component | GO:0005694 | chromosome | MCHK_RS12310  MCHK_RS07975 |  |
| Cellular Component | GO:0009380 | excinuclease repair complex | MCHK_RS11715 |  |
| Cellular Component | GO:0045277 | respiratory chain complex IV | MCHK_RS04655 |  |
| Molecular Function | GO:0003676 | nucleic acid binding | MCHK_RS04705 MCHK_RS06925  MCHK_RS13180  MCHK_RS33265 | MCHK_RS11735  MCHK_RS24605  MCHK_RS30800  MCHK_RS07470 |
| Molecular Function | GO:0003677 | DNA binding | MCHK_RS27730  MCHK_RS16665  MCHK_RS05195  MCHK_RS24460  MCHK_RS17330  MCHK_RS09850  MCHK_RS24600  MCHK_RS00370 MCHK_RS06080  MCHK_RS06200  MCHK_RS12310  MCHK_RS12935  MCHK_RS16965  MCHK_RS25705  MCHK_RS12300  MCHK_RS30950  MCHK_RS09830  MCHK_RS02590  MCHK_RS05190  MCHK_RS05295  MCHK_RS05365  MCHK_RS07285  MCHK_RS07290  MCHK_RS15555  MCHK_RS17475  MCHK_RS18365  MCHK_RS18900  MCHK_RS18935  MCHK_RS20525  MCHK_RS22290  MCHK_RS26640  MCHK_RS27460  MCHK_RS32205 | MCHK_RS33215  MCHK_RS05435  MCHK_RS06540  MCHK_RS06925  MCHK_RS07975  MCHK_RS09610  MCHK_RS13155  MCHK_RS16300  MCHK_RS18700  MCHK_RS21725  MCHK_RS21930  MCHK_RS24215  MCHK_RS25760  MCHK_RS27785  MCHK_RS29015  MCHK_RS29960  MCHK_RS32590  MCHK_RS01760  MCHK_RS15380  MCHK_RS31770  MCHK_RS27740  MCHK_RS23615  MCHK_RS22530  MCHK_RS31855  MCHK_RS27310  MCHK_RS13545  MCHK_RS26555  MCHK_RS30030  MCHK_RS20940  MCHK_RS23945  MCHK_RS19795  MCHK_RS05730  MCHK_RS32905 |
| Molecular Function | GO:0003723 | RNA binding | MCHK_RS31790  MCHK_RS25250  MCHK_RS04360  MCHK_RS22055  MCHK_RS00020 | MCHK_RS09895  MCHK_RS28765  MCHK_RS05735  MCHK_RS30800 |
| Molecular Function | GO:0003824 | catalytic activity | MCHK_RS01515  MCHK_RS02025  MCHK_RS02850  MCHK_RS03555  MCHK_RS04975  MCHK_RS07025  MCHK_RS09015  MCHK_RS09175  MCHK_RS09240  MCHK_RS09630  MCHK_RS11185  MCHK_RS11370  MCHK_RS13015  MCHK_RS13490  MCHK_RS15740  MCHK_RS18125  MCHK_RS20100  MCHK_RS20335  MCHK_RS20540  MCHK_RS24905  MCHK_RS27520  MCHK_RS28160  MCHK_RS29140  MCHK_RS30440  MCHK_RS31680  MCHK_RS32620  MCHK_RS32910  MCHK_RS08900  MCHK_RS19055  MCHK_RS03510  MCHK_RS01905  MCHK_RS04295  MCHK_RS11285  MCHK_RS11295  MCHK_RS24245  MCHK_RS04755  MCHK_RS21050  MCHK_RS00325  MCHK_RS01310  MCHK_RS02005  MCHK_RS03375  MCHK_RS03910  MCHK_RS04200  MCHK_RS05175  MCHK_RS07670  MCHK_RS10375 | MCHK_RS16205  MCHK_RS18215  MCHK_RS20410  MCHK_RS21180  MCHK_RS23545  MCHK_RS24875  MCHK_RS27405  MCHK_RS27510  MCHK_RS28400  MCHK_RS30820  MCHK_RS32625  MCHK_RS32680  MCHK_RS00455  MCHK_RS13780  MCHK_RS23035  MCHK_RS10270  MCHK_RS02470  MCHK_RS30740  MCHK_RS33230  MCHK_RS01740  MCHK_RS03670  MCHK_RS00030  MCHK_RS09025  MCHK_RS01215  MCHK_RS07615  MCHK_RS09205  MCHK_RS15605  MCHK_RS16495  MCHK_RS16865  MCHK_RS17900  MCHK_RS20080  MCHK_RS27965  MCHK_RS01815  MCHK_RS03815  MCHK_RS03955  MCHK_RS04580  MCHK_RS05015  MCHK_RS09725  MCHK_RS10540  MCHK_RS13930  MCHK_RS25060  MCHK_RS27765  MCHK_RS29070  MCHK_RS29190  MCHK_RS26130  MCHK_RS21930 |
| Molecular Function | GO:0003855 | 3-dehydroquinate dehydratase activity | MCHK_RS09440 |  |
| Molecular Function | GO:0003993 | acid phosphatase activity | MCHK_RS20065 |  |
| Molecular Function | GO:0004040 | amidase activity | MCHK_RS21965 |  |
| Molecular Function | GO:0004797 | thymidine kinase activity | MCHK_RS20240 |  |
| Molecular Function | GO:0004970 | ionotropic glutamate receptor activity | MCHK_RS17480 |  |
| Molecular Function | GO:0005267 | potassium channel activity | MCHK_RS22870 |  |
| Molecular Function | GO:0005488 | binding | MCHK_RS01315 |  |
| Molecular Function | GO:0005509 | calcium ion binding | MCHK_RS16435 |  |
| Molecular Function | GO:0005515 | protein binding | MCHK_RS18230  MCHK_RS01355  MCHK_RS05505  MCHK_RS07875  MCHK_RS16960  MCHK_RS18185  MCHK_RS18230  MCHK_RS20395  MCHK_RS21580  MCHK_RS25615  MCHK_RS27035  MCHK_RS27035 | MCHK_RS28655  MCHK_RS31455  MCHK_RS23255  MCHK_RS04740  MCHK_RS01760  MCHK_RS09485  MCHK_RS16805  MCHK_RS29775  MCHK_RS21725  MCHK_RS19795  MCHK_RS05730 |
| Molecular Function | GO:0005524 | ATP binding | MCHK_RS01150  MCHK_RS01450  MCHK_RS01480  MCHK_RS01985  MCHK_RS03240  MCHK_RS03570  MCHK_RS04310  MCHK_RS05225  MCHK_RS06960  MCHK_RS07295  MCHK_RS07325  MCHK_RS11390  MCHK_RS12705  MCHK_RS13815  MCHK_RS14685  MCHK_RS15825  MCHK_RS16880  MCHK_RS22580  MCHK_RS26715  MCHK_RS27340  MCHK_RS28060  MCHK_RS28085  MCHK_RS28780  MCHK_RS31965  MCHK_RS25105  MCHK_RS00060  MCHK_RS03715  MCHK_RS04340  MCHK_RS07250  MCHK_RS08520 | MCHK_RS11975  MCHK_RS16770  MCHK_RS17130  MCHK_RS22505  MCHK_RS22690  MCHK_RS28965  MCHK_RS30555  MCHK_RS30555  MCHK_RS33025  MCHK_RS06080  MCHK_RS13930  MCHK_RS01550  MCHK_RS12310  MCHK_RS11850  MCHK_RS28870  MCHK_RS12425  MCHK_RS28920  MCHK_RS24245  MCHK_RS17125  MCHK_RS20935  MCHK_RS11230  MCHK_RS29680  MCHK_RS07975  MCHK_RS15055  MCHK_RS14005  MCHK_RS19795  MCHK_RS32065  MCHK_RS00305  MCHK_RS12960  MCHK_RS11105 |
| Molecular Function | GO:0005525 | GTP binding | MCHK_RS25990  MCHK_RS24985 | MCHK_RS30040  MCHK_RS19490 |
| Molecular Function | GO:0005975 | carbohydrate metabolic process | MCHK_RS01905  MCHK_RS33025  MCHK_RS05015  MCHK_RS15530 | MCHK_RS04295  MCHK_RS29070  MCHK_RS25640 |
| Molecular Function | GO:0001510 | RNA methylation | MCHK_RS07925 |  |
| Molecular Function | GO:0008171 | O-methyltransferase activity | MCHK_RS09835 |  |
| Molecular Function | GO:0008270 | zinc ion binding | MCHK_RS11645  MCHK_RS28080  MCHK_RS05295 | MCHK_RS12080  MCHK_RS23945  MCHK_RS11105 |
| Molecular Function | GO:0016491 | oxidoreductase activity | MCHK_RS30380  MCHK_RS01460  MCHK_RS00650  MCHK_RS02110  MCHK_RS02045  MCHK_RS03150  MCHK_RS14890  MCHK_RS12360  MCHK_RS03150  MCHK_RS27470  MCHK_RS24135  MCHK_RS22980  MCHK_RS18130  MCHK_RS27470  MCHK_RS12320  MCHK_RS16755  MCHK_RS31370  MCHK_RS00975  MCHK_RS02150  MCHK_RS03310  MCHK_RS07080  MCHK_RS07220  MCHK_RS08050  MCHK_RS10330  MCHK_RS11605  MCHK_RS17325  MCHK_RS19290  MCHK_RS21205  MCHK_RS22175  MCHK_RS24405  MCHK_RS26860  MCHK_RS28600  MCHK_RS29450  MCHK_RS29820 | MCHK_RS01270  MCHK_RS01500  MCHK_RS04175  MCHK_RS10570  MCHK_RS11745  MCHK_RS15570  MCHK_RS15605  MCHK_RS16210  MCHK_RS16495  MCHK_RS17900  MCHK_RS18025  MCHK_RS19700  MCHK_RS21415  MCHK_RS27120  MCHK_RS27965  MCHK_RS28080  MCHK_RS28530  MCHK_RS29365  MCHK_RS01970  MCHK_RS14085  MCHK_RS04720  MCHK_RS04000  MCHK_RS21765  MCHK_RS13475  MCHK_RS01215  MCHK_RS07615  MCHK_RS09205  MCHK_RS16865  MCHK_RS20080  MCHK_RS26030  MCHK_RS13320  MCHK_RS25640  MCHK_RS13295 |
| Molecular Function | GO:0016740 | transferase activity | MCHK_RS00240  MCHK_RS33510  MCHK_RS00790  MCHK_RS28635 | MCHK_RS09895  MCHK_RS00030  MCHK_RS09025  MCHK_RS10540 |
| Molecular Function | GO:0016757 | transferase activity, transferring glycosyl groups | MCHK_RS00290 |  |
| Molecular Function | GO:0016773 | phosphotransferase activity, alcohol group as acceptor | MCHK_RS23140  MCHK_RS03575 | MCHK_RS04980 |
| Molecular Function | GO:0016787 | hydrolase activity | MCHK_RS01510  MCHK_RS04315  MCHK_RS07690  MCHK_RS12870  MCHK_RS13450  MCHK_RS15240  MCHK_RS15240  MCHK_RS15660  MCHK_RS16315  MCHK_RS16315  MCHK_RS23490  MCHK_RS24570  MCHK_RS24585 | MCHK_RS26460  MCHK_RS30580  MCHK_RS30605  MCHK_RS04775  MCHK_RS06405  MCHK_RS10995  MCHK_RS11265  MCHK_RS20295  MCHK_RS27420  MCHK_RS27510  MCHK_RS28375  MCHK_RS19795 |
| Molecular Function | GO:0016793 | triphosphoric monoester hydrolase activity | MCHK_RS12915 |  |
| Molecular Function | GO:0016799 | hydrolase activity, hydrolyzing N-glycosyl compounds | MCHK_RS21980 | MCHK_RS16765 |
| Molecular Function | GO:0016810 | hydrolase activity, acting on carbon-nitrogen (but not peptide) bonds | MCHK_RS13805  MCHK_RS20695  MCHK_RS02845  MCHK_RS26460  MCHK_RS29070  MCHK_RS11265  MCHK_RS27420  MCHK_RS20295  MCHK_RS03715 | MCHK_RS08520  MCHK_RS11975  MCHK_RS22505  MCHK_RS22690  MCHK_RS28965  MCHK_RS11850  MCHK_RS28375  MCHK_RS28020 |
| Molecular Function | GO:0016829 | lyase activity | MCHK_RS23575  MCHK_RS03555  MCHK_RS22375 | MCHK_RS21180  MCHK_RS24875 |
| Molecular Function | GO:0008690 | 3-deoxy-manno-octulosonate cytidylyltransferase activity | MCHK_RS30565 |  |
| Molecular Function | GO:0008830 | dTDP-4-dehydrorhamnose 3,5-epimerase activity | MCHK_RS04855 |  |
| Molecular Function | GO:0008838 | diaminopropionate ammonia-lyase activity | MCHK_RS09470 |  |
| Molecular Function | GO:0009055 | electron carrier activity | MCHK_RS01200  MCHK_RS01200  MCHK_RS04655  MCHK_RS08840 | MCHK_RS01500  MCHK_RS04175  MCHK_RS16210 |
| Molecular Function | GO:0010181 | FMN binding | MCHK_RS12185  MCHK_RS01215  MCHK_RS07615  MCHK_RS09205 | MCHK_RS14075  MCHK_RS16865  MCHK_RS20080  MCHK_RS30230 |
| Molecular Function | GO:0015035 | protein disulfide oxidoreductase activity | MCHK_RS10590 | MCHK_RS08840 |
| Molecular Function | GO:0051537 | 2 iron, 2 sulfur cluster binding | MCHK_RS27265  MCHK_RS01500 | MCHK_RS04175 |
| Molecular Function | GO:0051539 | 4 iron, 4 sulfur cluster binding | MCHK_RS14075  MCHK_RS10540  MCHK_RS10270 | MCHK_RS30230  MCHK_RS22375 |
| Molecular Function | GO:0000104 | succinate dehydrogenase activity | MCHK_RS25795 |  |
| Molecular Function | GO:0000166 | nucleotide binding | MCHK_RS29930  MCHK_RS30555  MCHK_RS00305  MCHK_RS01700 | MCHK_RS12960  MCHK_RS14005  MCHK_RS25105  MCHK_RS11105 |
| Molecular Function | GO:0000155 | phosphorelay sensor kinase activity | MCHK_RS04740  MCHK_RS16805  MCHK_RS17825  MCHK_RS10290  MCHK_RS02120  MCHK_RS06445  MCHK_RS16030 | MCHK_RS17305  MCHK_RS23255  MCHK_RS26340  MCHK_RS26490  MCHK_RS29680  MCHK_RS29855 |
| Molecular Function | GO:0000906 | 6,7-dimethyl-8-ribityllumazine synthase activity | MCHK_RS03850 |  |
| Molecular Function | GO:0003697 | single-stranded DNA binding | MCHK_RS11525 |  |
| Molecular Function | GO:0003700 | transcription factor activity, sequence-specific DNA binding | MCHK_RS00385  MCHK_RS01625  MCHK_RS01940  MCHK_RS03230  MCHK_RS03805  MCHK_RS05405  MCHK_RS05495  MCHK_RS05895  MCHK_RS05905  MCHK_RS05965  MCHK_RS07115  MCHK_RS07570  MCHK_RS08040  MCHK_RS09125  MCHK_RS10300  MCHK_RS14585  MCHK_RS15580  MCHK_RS16155  MCHK_RS16660  MCHK_RS17300  MCHK_RS17350  MCHK_RS19250  MCHK_RS19510  MCHK_RS19675  MCHK_RS20775 | MCHK_RS23165  MCHK_RS27805  MCHK_RS30525  MCHK_RS33020  MCHK_RS02590  MCHK_RS05365  MCHK_RS07285  MCHK_RS08900  MCHK_RS17475  MCHK_RS18365  MCHK_RS18900  MCHK_RS19055  MCHK_RS20525  MCHK_RS26640  MCHK_RS27460  MCHK_RS16300  MCHK_RS18700  MCHK_RS21930  MCHK_RS24215  MCHK_RS27785  MCHK_RS29015  MCHK_RS29960  MCHK_RS23945  MCHK_RS13295 |
| Molecular Function | GO:0003747 | translation release factor activity | MCHK_RS22765 |  |
| Molecular Function | GO:0003856 | 3-dehydroquinate synthase activity | MCHK_RS23285 |  |
| Molecular Function | GO:0003924 | GTPase activity | MCHK_RS24985 | MCHK_RS30040 |
| Molecular Function | GO:0003951 | NAD+ kinase activity | MCHK_RS18780  MCHK_RS23805 | MCHK_RS09560 |
| Molecular Function | GO:0003952 | NAD+ synthase (glutamine-hydrolyzing) activity | MCHK_RS04340 | MCHK_RS11850 |
| Molecular Function | GO:0003989 | acetyl-CoA carboxylase activity | MCHK_RS09425 |  |
| Molecular Function | GO:0004129 | cytochrome-c oxidase activity | MCHK_RS04720  MCHK_RS12785 | MCHK_RS04655 |
| Molecular Function | GO:0004151 | dihydroorotase activity | MCHK_RS11265 |  |
| Molecular Function | GO:0004177 | aminopeptidase activity | MCHK_RS04265 |  |
| Molecular Function | GO:0004190 | aspartic-type endopeptidase activity | MCHK_RS10780 |  |
| Molecular Function | GO:0004197 | cysteine-type endopeptidase activity | MCHK_RS29775 |  |
| Molecular Function | GO:0004222 | metalloendopeptidase activity | MCHK_RS30730  MCHK_RS09485 | MCHK_RS21670  MCHK_RS04755 |
| Molecular Function | GO:0004252 | serine-type endopeptidase activity | MCHK_RS02125  MCHK_RS05280 | MCHK_RS20445  MCHK_RS22235 |
| Molecular Function | GO:0004324 | ferredoxin-NADP+ reductase activity | MCHK_RS11745 |  |
| Molecular Function | GO:0004358 | glutamate N-acetyltransferase activity | MCHK_RS22850 |  |
| Molecular Function | GO:0004371 | glycerone kinase activity | MCHK_RS29790 |  |
| Molecular Function | GO:0004401 | histidinol-phosphatase activity | MCHK_RS21280 |  |
| Molecular Function | GO:0004413 | homoserine kinase activity | MCHK_RS04715 |  |
| Molecular Function | GO:0004427 | inorganic diphosphatase activity | MCHK_RS08180 |  |
| Molecular Function | GO:0004497 | monooxygenase activity | MCHK_RS03920 |  |
| Molecular Function | GO:0004523 | RNA-DNA hybrid ribonuclease activity | MCHK_RS04705 | MCHK_RS04360 |
| Molecular Function | GO:0004611 | phosphoenolpyruvate carboxykinase activity | MCHK_RS28920 |  |
| Molecular Function | GO:0004616 | phosphogluconate dehydrogenase (decarboxylating) activity | MCHK_RS13475  MCHK_RS15570 | MCHK_RS19700 |
| Molecular Function | GO:0004641 | phosphoribosylformylglycinamidine cyclo-ligase activity | MCHK_RS06500 |  |
| Molecular Function | GO:0004719 | protein-L-isoaspartate (D-aspartate) O-methyltransferase activity | MCHK_RS12860 |  |
| Molecular Function | GO:0004725 | protein tyrosine phosphatase activity | MCHK_RS26255 | MCHK_RS13850 |
| Molecular Function | GO:0004826 | phenylalanine-tRNA ligase activity | MCHK_RS31790 |  |
| Molecular Function | GO:0005198 | structural molecule activity | MCHK_RS20830 |  |
| Molecular Function | GO:0005524 | ATP binding | MCHK_RS24440  MCHK_RS31755  MCHK_RS26280  MCHK_RS24460  MCHK_RS20240  MCHK_RS11715  MCHK_RS29945 | MCHK_RS27385  MCHK_RS00840  MCHK_RS18135  MCHK_RS12390  MCHK_RS13985  MCHK_RS22000 |
| Cellular Component | GO:0005737 | cytoplasm | MCHK_RS30565  MCHK_RS11105  MCHK_RS00480  MCHK_RS06500  MCHK_RS12780  MCHK_RS24955  MCHK_RS20160  MCHK_RS07250  MCHK_RS30040  MCHK_RS22765 | MCHK_RS23285  MCHK_RS11110  MCHK_RS28170  MCHK_RS14005  MCHK_RS26130  MCHK_RS10540  MCHK_RS00305  MCHK_RS12960  MCHK_RS15055  MCHK_RS32065 |
| Molecular Function | GO:0008081 | phosphoric diester hydrolase activity | MCHK_RS08405 | MCHK_RS10180 |
| Molecular Function | GO:0008113 | peptide-methionine (S)-S-oxide reductase activity | MCHK_RS15845 |  |
| Molecular Function | GO:0008115 | sarcosine oxidase activity | MCHK_RS02875  MCHK_RS04000 | MCHK_RS21765 |
| Molecular Function | GO:0008137 | NADH dehydrogenase (ubiquinone) activity | MCHK_RS14075  MCHK_RS30230 | MCHK_RS14025 |
| Molecular Function | GO:0008233 | peptidase activity | MCHK_RS04375  MCHK_RS32985 | MCHK_RS11230 |
| Molecular Function | GO:0008237 | metallopeptidase activity | MCHK_RS04635 |  |
| Molecular Function | GO:0008272 | sulfate transport | MCHK_RS15485 |  |
| Molecular Function | GO:0008410 | CoA-transferase activity | MCHK_RS25465 |  |
| Molecular Function | GO:0008425 | 2-polyprenyl-6-methoxy-1,4-benzoquinone methyltransferase activity | MCHK_RS22775 |  |
| Molecular Function | GO:0008444 | CDP-diacylglycerol-glycerol-3-phosphate 3-phosphatidyltransferase activity | MCHK_RS18745 |  |
| Molecular Function | GO:0008448 | N-acetylglucosamine-6-phosphate deacetylase activity | MCHK_RS27420 |  |
| Molecular Function | GO:0008478 | pyridoxal kinase activity | MCHK_RS25315 |  |
| Molecular Function | GO:0008483 | transaminase activity | MCHK_RS13490  MCHK_RS18125 | MCHK_RS30740 |
| Molecular Function | GO:0008495 | protoheme IX farnesyltransferase activity | MCHK_RS12755 |  |
| Molecular Function | GO:0008556 | potassium-transporting ATPase activity | MCHK_RS21620 |  |
| Molecular Function | GO:0008565 | protein transporter activity | MCHK_RS20755  MCHK_RS24745 | MCHK_RS07070 |
| Molecular Function | GO:0008671 | 2-dehydro-3-deoxygalactonokinase activity | MCHK_RS27325 |  |
| Molecular Function | GO:0008716 | D-alanine-D-alanine ligase activity | MCHK_RS27340 |  |
| Molecular Function | GO:0008759 | UDP-3-O-[3-hydroxymyristoyl] N-acetylglucosamine deacetylase activity | MCHK_RS15015 |  |
| Molecular Function | GO:0008773 | [protein-PII] uridylyltransferase activity | MCHK_RS29950 |  |
| Molecular Function | GO:0008794 | arsenate reductase (glutaredoxin) activity | MCHK_RS17290 |  |
| Molecular Function | GO:0008818 | cobalamin 5'-phosphate synthase activity | MCHK_RS14170 |  |
| Molecular Function | GO:0008914 | leucyltransferase activity | MCHK_RS09420 |  |
| Molecular Function | GO:0008915 | lipid-A-disaccharide synthase activity | MCHK_RS08730 |  |
| Molecular Function | GO:0008976 | polyphosphate kinase activity | MCHK_RS00245 |  |
| Molecular Function | GO:0009039 | urease activity | MCHK_RS28375 |  |
| Molecular Function | GO:0009041 | uridylate kinase activity | MCHK_RS11110 |  |
| Molecular Function | GO:0015079 | potassium ion transmembrane transporter activity | MCHK_RS02780 |  |
| Molecular Function | GO:0015099 | nickel cation transmembrane transporter activity | MCHK_RS21080 |  |
| Molecular Function | GO:0015159 | polysaccharide transmembrane transporter activity | MCHK_RS08415 |  |
| Molecular Function | GO:0015225 | biotin transporter activity | MCHK_RS05005 |  |
| Molecular Function | GO:0015288 | porin activity | MCHK_RS05680 |  |
| Molecular Function | GO:0015716 | organic phosphonate transport | MCHK_RS22410 |  |
| Molecular Function | GO:0015888 | thiamine transport | MCHK_RS24450 |  |
| Molecular Function | GO:0016616 | oxidoreductase activity, acting on the CH-OH group of donors, NAD or NADP as acceptor | MCHK_RS01275  MCHK_RS25640 | MCHK_RS33110 |
| Molecular Function | GO:0016705 | oxidoreductase activity, acting on paired donors, with incorporation or reduction of molecular oxygen | MCHK_RS32395  MCHK_RS33615 | MCHK_RS05175 |
| Molecular Function | GO:0016746 | transferase activity, transferring acyl groups | MCHK_RS12200 |  |
| Molecular Function | GO:0016779 | nucleotidyltransferase activity | MCHK_RS29745  MCHK_RS25060 | MCHK_RS29950  MCHK_RS05495 |
| Molecular Function | GO:0016790 | thiolester hydrolase activity | MCHK_RS24570 |  |
| Molecular Function | GO:0016836 | hydro-lyase activity | MCHK_RS12780 |  |
| Molecular Function | GO:0016846 | carbon-sulfur lyase activity | MCHK_RS09060  MCHK_RS11995 | MCHK_RS28835  MCHK_RS12080 |
| Molecular Function | GO:0016857 | racemase and epimerase activity, acting on carbohydrates and derivatives | MCHK_RS00480 | MCHK_RS01905 |
| Molecular Function | GO:0016887 | ATPase activity | MCHK_RS01150  MCHK_RS01450  MCHK_RS01480  MCHK_RS01985  MCHK_RS03240  MCHK_RS03570  MCHK_RS04310  MCHK_RS05225  MCHK_RS06960  MCHK_RS07295  MCHK_RS07325  MCHK_RS11390  MCHK_RS12705  MCHK_RS13815  MCHK_RS14685  MCHK_RS15825  MCHK_RS16880  MCHK_RS22580  MCHK_RS26715  MCHK_RS28060  MCHK_RS28085 | MCHK_RS28780  MCHK_RS17130  MCHK_RS12390  MCHK_RS13985  MCHK_RS22000  MCHK_RS24460  MCHK_RS30555  MCHK_RS30555  MCHK_RS11715  MCHK_RS24440  MCHK_RS31755  MCHK_RS03715  MCHK_RS08520  MCHK_RS11975  MCHK_RS22505  MCHK_RS22690  MCHK_RS28965  MCHK_RS11230  MCHK_RS32065  MCHK_RS17125  MCHK_RS20935 |
| Molecular Function | GO:0018738 | S-formylglutathione hydrolase activity | MCHK_RS02970 |  |
| Molecular Function | GO:0018822 | nitrile hydratase activity | MCHK_RS14530 |  |
| Molecular Function | GO:0019239 | deaminase activity | MCHK_RS16120 |  |
| Molecular Function | GO:0019808 | polyamine binding | MCHK_RS16815 |  |
| Molecular Function | GO:0030151 | molybdenum ion binding | MCHK_RS31680 | MCHK_RS14725 |
| Molecular Function | GO:0030170 | pyridoxal phosphate binding | MCHK_RS32910  MCHK_RS26985  MCHK_RS09470  MCHK_RS00455  MCHK_RS13490  MCHK_RS13780 | MCHK_RS18125  MCHK_RS31680  MCHK_RS08900  MCHK_RS19055  MCHK_RS30740 |
| Molecular Function | GO:0030976 | thiamine pyrophosphate binding | MCHK_RS30440 | MCHK_RS09725 |
| Molecular Function | GO:0031419 | cobalamin binding | MCHK_RS09630 | MCHK_RS27765 |
| Molecular Function | GO:0033743 | peptide-methionine (R)-S-oxide reductase activity | MCHK_RS27745 |  |
| Molecular Function | GO:0033863 | ribose 1,5-bisphosphate phosphokinase activity | MCHK_RS28545 |  |
| Molecular Function | GO:0036361 | racemase activity, acting on amino acids and derivatives | MCHK_RS08350 |  |
| Molecular Function | GO:0042026 | protein refolding | MCHK_RS07250 |  |
| Molecular Function | GO:0042626 | ATPase activity, coupled to transmembrane movement of substances | MCHK_RS07705  MCHK_RS17130 | MCHK_RS11230 |
| Molecular Function | GO:0043022 | ribosome binding | MCHK_RS25885 |  |
| Molecular Function | GO:0043190 | ATP-binding cassette (ABC) transporter complex | MCHK_RS00500  MCHK_RS04060  MCHK_RS26800  MCHK_RS30430  MCHK_RS30560  MCHK_RS07705  MCHK_RS06140  MCHK_RS22000 | MCHK_RS03715  MCHK_RS08520  MCHK_RS11975  MCHK_RS22505  MCHK_RS22690  MCHK_RS28965  MCHK_RS17125  MCHK_RS20935 |
| Molecular Function | GO:0043565 | sequence-specific DNA binding | MCHK_RS13545  MCHK_RS15380  MCHK_RS17330  MCHK_RS20940  MCHK_RS27740  MCHK_RS31770  MCHK_RS31855  MCHK_RS21930  MCHK_RS05495 | MCHK_RS01760  MCHK_RS01940  MCHK_RS10300  MCHK_RS19510  MCHK_RS07285  MCHK_RS17475  MCHK_RS18900  MCHK_RS20525  MCHK_RS26640 |
| Molecular Function | GO:0043772 | acyl-phosphate glycerol-3-phosphate acyltransferase activity | MCHK_RS11270 |  |
| Molecular Function | GO:0046872 | metal ion binding | MCHK_RS30580  MCHK_RS28020  MCHK_RS09630  MCHK_RS21080  MCHK_RS27340  MCHK_RS04755 | MCHK_RS01500  MCHK_RS24245  MCHK_RS11265  MCHK_RS01700  MCHK_RS27765 |
| Molecular Function | GO:0046912 | transferase activity, transferring acyl groups, acyl groups converted into alkyl on transfer | MCHK_RS24350 |  |
| Molecular Function | GO:0048472 | threonine-phosphate decarboxylase activity | MCHK_RS13855 |  |
| Molecular Function | GO:0050662 | coenzyme binding | MCHK_RS01905  MCHK_RS02850 | MCHK_RS13015 |
| Molecular Function | GO:0051287 | NAD binding | MCHK_RS03055  MCHK_RS30530  MCHK_RS32065  MCHK_RS25640  MCHK_RS13475  MCHK_RS14075 | MCHK_RS01275  MCHK_RS15570  MCHK_RS19700  MCHK_RS33110  MCHK_RS22130 |
| Molecular Function | GO:0051920 | peroxiredoxin activity | MCHK_RS15385 | MCHK_RS17545 |
| Molecular Function | GO:0052906 | tRNA (guanine(37)-N(1))-methyltransferase activity | MCHK_RS25895 |  |
| Molecular Function | GO:0097367 | carbohydrate derivative binding | MCHK_RS08490  MCHK_RS19525 | MCHK_RS27430  MCHK_RS16300 |
| Molecular Function | GO:0000049 | tRNA binding | MCHK_RS11105 | MCHK_RS00305 |
| Molecular Function | GO:0000175 | 3'-5'-exoribonuclease activity | MCHK_RS30800 |  |
| Molecular Function | GO:0003678 | DNA helicase activity | MCHK_RS06080 |  |
| Molecular Function | GO:0003743 | translation initiation factor activity | MCHK_RS28765 |  |
| Molecular Function | GO:0003755 | peptidyl-prolyl cis-trans isomerase activity | MCHK_RS11470 |  |
| Molecular Function | GO:0003796 | lysozyme activity | MCHK_RS15180 |  |
| Molecular Function | GO:0003857 | 3-hydroxyacyl-CoA dehydrogenase activity | MCHK_RS01970 | MCHK_RS14085 |
| Molecular Function | GO:0003864 | 3-methyl-2-oxobutanoate hydroxymethyltransferase activity | MCHK_RS33230 |  |
| Molecular Function | GO:0003867 | 4-aminobutyrate transaminase activity | MCHK_RS30740 |  |
| Molecular Function | GO:0003887 | DNA-directed DNA polymerase activity | MCHK_RS06200 |  |
| Molecular Function | GO:0003899 | DNA-directed 5'-3' RNA polymerase activity | MCHK_RS09830 |  |
| Molecular Function | GO:0003910 | DNA ligase (ATP) activity | MCHK_RS01550 |  |
| Molecular Function | GO:0003918 | DNA topoisomerase type II (ATP-hydrolyzing) activity | MCHK_RS07975 | MCHK_RS12310 |
| Molecular Function | GO:0003999 | adenine phosphoribosyltransferase activity | MCHK_RS20160 |  |
| Molecular Function | GO:0004072 | aspartate kinase activity | MCHK_RS22750 |  |
| Molecular Function | GO:0004096 | catalase activity | MCHK_RS02445 |  |
| Molecular Function | GO:0004326 | tetrahydrofolylpolyglutamate synthase activity | MCHK_RS28870 | MCHK_RS15055 |
| Molecular Function | GO:0004340 | glucokinase activity | MCHK_RS12425 |  |
| Molecular Function | GO:0004518 | nuclease activity | MCHK_RS12935  MCHK_RS16965 | MCHK_RS25705 |
| Molecular Function | GO:0004601 | peroxidase activity | MCHK_RS21030 | MCHK_RS02445 |
| Molecular Function | GO:0004612 | phosphoenolpyruvate carboxykinase (ATP) activity | MCHK_RS28920 |  |
| Molecular Function | GO:0004638 | phosphoribosylaminoimidazole carboxylase activity | MCHK_RS24245 |  |
| Molecular Function | GO:0004803 | transposase activity | MCHK_RS12300 | MCHK_RS30950 |
| Molecular Function | GO:0004871 | signal transducer activity | MCHK_RS17825  MCHK_RS06445 | MCHK_RS26340  MCHK_RS26490 |
| Molecular Function | GO:0005215 | transporter activity | MCHK_RS00405  MCHK_RS01425  MCHK_RS03305  MCHK_RS03715  MCHK_RS04185  MCHK_RS05210  MCHK_RS05870  MCHK_RS06205  MCHK_RS07105  MCHK_RS08520  MCHK_RS11975  MCHK_RS15590  MCHK_RS16470  MCHK_RS17490  MCHK_RS17490  MCHK_RS19340  MCHK_RS22505 | MCHK_RS22690  MCHK_RS24115  MCHK_RS24195  MCHK_RS27490  MCHK_RS27515  MCHK_RS28290  MCHK_RS28965  MCHK_RS30650  MCHK_RS02075  MCHK_RS17810  MCHK_RS25470  MCHK_RS15685  MCHK_RS17125  MCHK_RS19655  MCHK_RS20935  MCHK_RS24450 |
| Molecular Function | GO:0005216 | ion channel activity | MCHK_RS26135 |  |
| Molecular Function | GO:0005507 | copper ion binding | MCHK_RS04720  MCHK_RS04655  MCHK_RS29960 | MCHK_RS12595  MCHK_RS12785 |
| Molecular Function | GO:0008097 | 5S rRNA binding | MCHK_RS20030 |  |
| Molecular Function | GO:0008236 | serine-type peptidase activity | MCHK_RS05280 | MCHK_RS22235 |
| Molecular Function | GO:0008324 | cation transmembrane transporter activity | MCHK_RS21090 | MCHK_RS11680 |
| Molecular Function | GO:0008442 | 3-hydroxyisobutyrate dehydrogenase activity | MCHK_RS13475 |  |
| Molecular Function | GO:0008703 | 5-amino-6-(5-phosphoribosylamino)uracil reductase activity | MCHK_RS16280 |  |
| Molecular Function | GO:0008760 | UDP-N-acetylglucosamine 1-carboxyvinyltransferase activity | MCHK_RS01740 | MCHK_RS03670 |
| Molecular Function | GO:0008836 | diaminopimelate decarboxylase activity | MCHK_RS23035 |  |
| Molecular Function | GO:0008882 | [glutamate-ammonia-ligase] adenylyltransferase activity | MCHK_RS05495 |  |
| Molecular Function | GO:0008889 | glycerophosphodiester phosphodiesterase activity | MCHK_RS08405 |  |
| Molecular Function | GO:0008934 | inositol monophosphate 1-phosphatase activity | MCHK_RS24670 |  |
| Molecular Function | GO:0009381 | excinuclease ABC activity | MCHK_RS11715 | MCHK_RS19795 |
| Molecular Function | GO:0009678 | hydrogen-translocating pyrophosphatase activity | MCHK_RS08180 |  |
| Molecular Function | GO:0015036 | disulfide oxidoreductase activity | MCHK_RS26030 | MCHK_RS08840 |
| Molecular Function | GO:0015116 | sulfate transmembrane transporter activity | MCHK_RS15485 |  |
| Molecular Function | GO:0015232 | heme transporter activity | MCHK_RS07765 |  |
| Molecular Function | GO:0015424 | amino acid-transporting ATPase activity | MCHK_RS24440 | MCHK_RS31755 |
| Molecular Function | GO:0015604 | organic phosphonate transmembrane transporter activity | MCHK_RS22410 |  |
| Molecular Function | GO:0016149 | translation release factor activity, codon specific | MCHK_RS22765 |  |
| Molecular Function | GO:0016151 | nickel cation binding | MCHK_RS28375 | MCHK_RS28170 |
| Molecular Function | GO:0016407 | acetyltransferase activity | MCHK_RS30725 |  |
| Molecular Function | GO:0016614 | oxidoreductase activity, acting on CH-OH group of donors | MCHK_RS11605  MCHK_RS15605  MCHK_RS16495 | MCHK_RS17900  MCHK_RS27965  MCHK_RS29820 |
| Molecular Function | GO:0016624 | oxidoreductase activity, acting on the aldehyde or oxo group of donors, disulfide as acceptor | MCHK_RS28400 |  |
| Molecular Function | GO:0016627 | oxidoreductase activity, acting on the CH-CH group of donors | MCHK_RS21475  MCHK_RS25795 | MCHK_RS30365 |
| Molecular Function | GO:0016765 | transferase activity, transferring alkyl or aryl (other than methyl) groups | MCHK_RS12755  MCHK_RS01740 | MCHK_RS03670 |
| Molecular Function | GO:0016780 | phosphotransferase activity, for other substituted phosphate groups | MCHK_RS18745 |  |
| Molecular Function | GO:0016791 | phosphatase activity | MCHK_RS13850 |  |
| Molecular Function | GO:0016813 | hydrolase activity, acting on carbon-nitrogen (but not peptide) bonds, in linear amidines | MCHK_RS06405 |  |
| Molecular Function | GO:0016838 | carbon-oxygen lyase activity, acting on phosphates | MCHK_RS23285 |  |
| Molecular Function | GO:0016992 | lipoate synthase activity | MCHK_RS10270 |  |
| Molecular Function | GO:0019829 | cation-transporting ATPase activity | MCHK_RS01700 | MCHK_RS29930 |
| Molecular Function | GO:0019843 | rRNA binding | MCHK_RS09970 |  |
| Molecular Function | GO:0020037 | heme binding | MCHK_RS02445  MCHK_RS21030  MCHK_RS07765  MCHK_RS16210  MCHK_RS25795 | MCHK_RS32355  MCHK_RS33615  MCHK_RS04655  MCHK_RS22175 |
| Molecular Function | GO:0030983 | mismatched DNA binding | MCHK_RS29945 |  |
| Molecular Function | GO:0033862 | UMP kinase activity | MCHK_RS11110 |  |
| Molecular Function | GO:0042132 | fructose 1,6-bisphosphate 1-phosphatase activity | MCHK_RS12590 |  |
| Molecular Function | GO:0046914 | transition metal ion binding | MCHK_RS14530 |  |
| Molecular Function | GO:0048027 | mRNA 5'-UTR binding | MCHK_RS20910 |  |
| Molecular Function | GO:0050660 | flavin adenine dinucleotide binding | MCHK_RS28600  MCHK_RS15605  MCHK_RS16495  MCHK_RS17900  MCHK_RS11605 | MCHK_RS21475  MCHK_RS30365  MCHK_RS27965  MCHK_RS10570 |
| Molecular Function | GO:0051073 | adenosylcobinamide-GDP ribazoletransferase activity | MCHK_RS14170 |  |
| Molecular Function | GO:0051082 | unfolded protein binding | MCHK_RS27385 | MCHK_RS16770 |
| Molecular Function | GO:0071949 | FAD binding | MCHK_RS07080  MCHK_RS10570 | MCHK_RS17325 |
| Molecular Function | GO:0002161 | aminoacyl-tRNA editing activity | MCHK_RS12960 | MCHK_RS25105 |
| Molecular Function | GO:0003844 | 1,4-alpha-glucan branching enzyme activity | MCHK_RS05015 |  |
| Molecular Function | GO:0003916 | DNA topoisomerase activity | MCHK_RS07975 |  |
| Molecular Function | GO:0004146 | dihydrofolate reductase activity | MCHK_RS14640 |  |
| Molecular Function | GO:0004356 | glutamate-ammonia ligase activity | MCHK_RS03955 | MCHK_RS29190 |
| Molecular Function | GO:0004411 | homogentisate 1,2-dioxygenase activity | MCHK_RS07715 |  |
| Molecular Function | GO:0004519 | endonuclease activity | MCHK_RS12935  MCHK_RS16965 | MCHK_RS25705 |
| Molecular Function | GO:0004520 | endodeoxyribonuclease activity | MCHK_RS24605 |  |
| Molecular Function | GO:0004525 | ribonuclease III activity | MCHK_RS05735 |  |
| Molecular Function | GO:0004588 | orotate phosphoribosyltransferase activity | MCHK_RS05695 |  |
| Molecular Function | GO:0004672 | protein kinase activity | MCHK_RS29680 |  |
| Molecular Function | GO:0004673 | protein histidine kinase activity | MCHK_RS16030 | MCHK_RS17305 |
| Molecular Function | GO:0004807 | triose-phosphate isomerase activity | MCHK_RS03815 |  |
| Molecular Function | GO:0004812 | aminoacyl-tRNA ligase activity | MCHK_RS14005  MCHK_RS11105  MCHK_RS00305 | MCHK_RS12960  MCHK_RS25105 |
| Molecular Function | GO:0005247 | voltage-gated chloride channel activity | MCHK_RS26135 |  |
| Molecular Function | GO:0008173 | RNA methyltransferase activity | MCHK_RS22055 | MCHK_RS26130 |
| Molecular Function | GO:0008234 | cysteine-type peptidase activity | MCHK_RS29775 |  |
| Molecular Function | GO:0008661 | 1-deoxy-D-xylulose-5-phosphate synthase activity | MCHK_RS04580 |  |
| Molecular Function | GO:0008795 | NAD+ synthase activity | MCHK_RS04340 |  |
| Molecular Function | GO:0008974 | phosphoribulokinase activity | MCHK_RS33025 |  |
| Molecular Function | GO:0009013 | succinate-semialdehyde dehydrogenase [NAD(P)+] activity | MCHK_RS13320 |  |
| Molecular Function | GO:0009028 | tartronate-semialdehyde synthase activity | MCHK_RS09725 |  |
| Molecular Function | GO:0015171 | amino acid transmembrane transporter activity | MCHK_RS18490 |  |
| Molecular Function | GO:0015419 | ATPase-coupled sulfate transmembrane transporter activity | MCHK_RS15485 |  |
| Molecular Function | GO:0015542 | sugar efflux transmembrane transporter activity | MCHK_RS29750 |  |
| Molecular Function | GO:0016651 | oxidoreductase activity, acting on NAD(P)H | MCHK_RS14075 |  |
| Molecular Function | GO:0016709 | oxidoreductase activity, acting on paired donors, with incorporation or reduction of molecular oxygen, NAD(P)H as one donor, and incorporation of one atom of oxygen | MCHK_RS27765 | MCHK_RS10570 |
| Molecular Function | GO:0016772 | transferase activity, transferring phosphorus-containing groups | MCHK_RS10290  MCHK_RS06445  MCHK_RS26340 | MCHK_RS26490  MCHK_RS02120  MCHK_RS29855 |
| Molecular Function | GO:0016788 | hydrolase activity, acting on ester bonds | MCHK_RS01815 |  |
| Molecular Function | GO:0016844 | strictosidine synthase activity | MCHK_RS15685 |  |
| Molecular Function | GO:0032549 | ribonucleoside binding | MCHK_RS09830 |  |
| Molecular Function | GO:0042954 | lipoprotein transporter activity | MCHK_RS12390 | MCHK_RS13985 |
| Molecular Function | GO:0043819 | precorrin-6A synthase (deacetylating) activity | MCHK_RS14670 |  |
| Molecular Function | GO:0070008 | serine-type exopeptidase activity | MCHK_RS05280 |  |
| Molecular Function | GO:0004367 | glycerol-3-phosphate dehydrogenase [NAD+] activity | MCHK_RS25640 |  |
| Molecular Function | GO:0004553 | hydrolase activity, hydrolyzing O-glycosyl compounds | MCHK_RS05015 |  |
| Molecular Function | GO:0004827 | proline-tRNA ligase activity | MCHK_RS14005 |  |
| Molecular Function | GO:0005506 | iron ion binding | MCHK_RS03510  MCHK_RS32355 | MCHK_RS33615  MCHK_RS04655 |
| Molecular Function | GO:0015299 | solute:proton antiporter activity | MCHK_RS11680 |  |
| Molecular Function | GO:0015417 | polyamine-transporting ATPase activity | MCHK_RS17125 | MCHK_RS20935 |
| Molecular Function | GO:0016874 | ligase activity | MCHK_RS15055 | MCHK_RS28870 |
| Molecular Function | GO:0016987 | sigma factor activity | MCHK_RS18700  MCHK_RS24215  MCHK_RS27785 | MCHK_RS29015  MCHK_RS23945 |
| Molecular Function | GO:0017076 | purine nucleotide binding | MCHK_RS28920 |  |
| Molecular Function | GO:0030145 | manganese ion binding | MCHK_RS20295 |  |
| Molecular Function | GO:0043858 | arginine:ornithine antiporter activity | MCHK_RS18490 |  |
| Molecular Function | GO:0050661 | NADP binding | MCHK_RS14640 | MCHK_RS27765 |
| Molecular Function | GO:0051907 | S-(hydroxymethyl)glutathione synthase activity | MCHK_RS12080 |  |
| Molecular Function | GO:0070403 | NAD+ binding | MCHK_RS01970 | MCHK_RS14085 |
| Molecular Function | GO:0003908 | methylated-DNA-[protein]-cysteine S-methyltransferase activity | MCHK_RS21930 |  |
| Molecular Function | GO:0004654 | polyribonucleotide nucleotidyltransferase activity | MCHK_RS30800 |  |
| Molecular Function | GO:0004823 | leucine-tRNA ligase activity | MCHK_RS25105 |  |
| Molecular Function | GO:0004824 | lysine-tRNA ligase activity | MCHK_RS00305 |  |
| Molecular Function | GO:0004832 | valine-tRNA ligase activity | MCHK_RS12960 |  |
| Molecular Function | GO:0004851 | uroporphyrin-III C-methyltransferase activity | MCHK_RS22130 |  |
| Molecular Function | GO:0008199 | ferric iron binding | MCHK_RS03510 |  |
| Molecular Function | GO:0016820 | hydrolase activity, acting on acid anhydrides, catalyzing transmembrane movement of substances | MCHK_RS17125 | MCHK_RS20935 |
| Molecular Function | GO:0043169 | cation binding | MCHK_RS05015 |  |
| Molecular Function | GO:0003842 | 1-pyrroline-5-carboxylate dehydrogenase activity | MCHK_RS13295 |  |
| Molecular Function | GO:0004818 | glutamate-tRNA ligase activity | MCHK_RS11105 |  |
| Molecular Function | GO:0008764 | UDP-N-acetylmuramoylalanine-D-glutamate ligase activity | MCHK_RS15055 |  |
| Molecular Function | GO:0016702 | oxidoreductase activity, acting on single donors with incorporation of molecular oxygen, incorporation of two atoms of oxygen | MCHK_RS03510 |  |
| Molecular Function | GO:0004657 | proline dehydrogenase activity | MCHK_RS13295 |  |
| Molecular Function | GO:0018578 | protocatechuate 3,4-dioxygenase activity | MCHK_RS03510 |  |
| Molecular Function | GO:0043115 | precorrin-2 dehydrogenase activity | MCHK_RS22130 |  |
| Molecular Function | GO:0046961 | proton-transporting ATPase activity, rotational mechanism | MCHK_RS32065 |  |
| Molecular Function | GO:0051536 | iron-sulfur cluster binding | MCHK_RS29140  MCHK_RS32620  MCHK_RS01200  MCHK_RS26130  MCHK_RS04175  MCHK_RS27120 | MCHK_RS10270  MCHK_RS22175  MCHK_RS09630  MCHK_RS10540  MCHK_RS01500  MCHK_RS27765 |
| Molecular Function | GO:0009331 | glycerol-3-phosphate dehydrogenase complex | MCHK_RS25640 |  |
| Molecular Function | GO:0051266 | sirohydrochlorin ferrochelatase activity | MCHK_RS22130 |  |
| Molecular Function | GO:0016876 | ligase activity, forming aminoacyl-tRNA and related compounds | MCHK_RS11105 |  |
| Cellular Component | GO:0003735 | structural constituent of ribosome | MCHK_RS08475  MCHK_RS09885  MCHK_RS09890  MCHK_RS09930  MCHK_RS09940  MCHK_RS09965 | MCHK_RS09970  MCHK_RS11930  MCHK_RS20030  MCHK_RS24720  MCHK_RS00020  MCHK_RS09895 |
| Cellular Component | GO:0005737 | cytoplasm | MCHK_RS30565  MCHK_RS11105  MCHK_RS00480  MCHK_RS06500  MCHK_RS12780  MCHK_RS24955  MCHK_RS20160  MCHK_RS07250  MCHK_RS30040  MCHK_RS22765 | MCHK_RS23285  MCHK_RS11110  MCHK_RS28170  MCHK_RS14005  MCHK_RS26130  MCHK_RS10540  MCHK_RS00305  MCHK_RS12960  MCHK_RS15055  MCHK_RS32065 |
| Cellular Component | GO:0071555 | cell wall organization | MCHK_RS17305 |  |
| Cellular Component | GO:0005840 | ribosome | MCHK_RS08475  MCHK_RS09885  MCHK_RS09890  MCHK_RS09930  MCHK_RS09965  MCHK_RS11930 | MCHK_RS24720  MCHK_RS20030  MCHK_RS00020  MCHK_RS09970  MCHK_RS09895  MCHK_RS25885 |
| Cellular Component | GO:0005886 | plasma membrane | MCHK_RS25565  MCHK_RS26020  MCHK_RS23840  MCHK_RS28795  MCHK_RS21775 | MCHK_RS07070  MCHK_RS04655  MCHK_RS05005  MCHK_RS05555  MCHK_RS11270 |
| Cellular Component | GO:0009349 | riboflavin synthase complex | MCHK_RS03850 |  |
| Cellular Component | GO:0015934 | large ribosomal subunit | MCHK_RS09940 | MCHK_RS09895 |
| Cellular Component | GO:0030254 | protein secretion by the type III secretion system | MCHK_RS32065 |  |
| Cellular Component | GO:0005694 | chromosome | MCHK_RS12310  MCHK_RS07975 |  |
| Cellular Component | GO:0009380 | excinuclease repair complex | MCHK_RS11715 |  |
| Cellular Component | GO:0045277 | respiratory chain complex IV | MCHK_RS04655 |  |
